# Measuring cognitive effort without difficulty

**DOI:** 10.3758/s13415-023-01065-9

**Published:** 2023-02-07

**Authors:** Hugo Fleming, Oliver J. Robinson, Jonathan P. Roiser

**Affiliations:** 1 Institute of Cognitive Neuroscience, University College London, 17 Queen Square, London, WC1N 3AZ UK; 2 MRC Cognition and Brain Sciences Unit, University of Cambridge, 15 Chaucer Road, Cambridge, CB2 7EF UK

**Keywords:** Anhedonia, Cognitive effort, Computational psychiatry, Depression, Individual differences, New measures

## Abstract

An important finding in the cognitive effort literature has been that sensitivity to the costs of effort varies between individuals, suggesting that some people find effort more aversive than others. It has been suggested this may explain individual differences in other aspects of cognition; in particular that greater effort sensitivity may underlie some of the symptoms of conditions such as depression and schizophrenia. In this paper, we highlight a major problem with existing measures of cognitive effort that hampers this line of research, specifically the confounding of effort and difficulty. This means that behaviour thought to reveal effort costs could equally be explained by cognitive capacity, which influences the frequency of success and thereby the chance of obtaining reward. To address this shortcoming, we introduce a new test, the Number Switching Task (NST), specially designed such that difficulty will be unaffected by the effort manipulation and can easily be standardised across participants. In a large, online sample, we show that these criteria are met successfully and reproduce classic effort discounting results with the NST. We also demonstrate the use of Bayesian modelling with this task, producing behavioural parameters which can be associated with other measures, and report a preliminary association with the Need for Cognition scale.

**Supplementary Information:**

The online version contains supplementary material available at 10.3758/s13415-023-01065-9.

## Introduction

Cognitive effort, our ability to vary the depth of our engagement with a cognitive task, influences a raft of fundamental cognitive processes including attention (Kahneman, 1973), working memory (Westbrook et al., 2013), cognitive control (Braver, 2012; Shenhav et al., 2013) and decision-making (Ortega et al., 2015; Toplak et al., 2011). Consequently, there is substantial interest in both measuring cognitive effort and understanding the factors that determine when and how much effort is exerted in different situations. Unfortunately, cognitive effort is challenging to study for the very same reason—because it is so entangled with other processes, there is considerable potential for confounding, and attempts to measure cognitive effort must therefore be careful to isolate effort from other factors that may influence performance.

One particular problem—the conflation of effort and difficulty—has not to our knowledge been addressed. Current methods for studying cognitive effort involve assessing participants’ preferences for different cognitive tasks: avoidance of more demanding tasks is interpreted as evidence of underlying effort costs, and these are quantified by examining how participants trade off the demand against rewards (a phenomenon termed effort discounting; see Westbrook & Braver, 2015). However, more demanding tasks also may have lower rates of success, and therefore of obtaining reward, giving rise to another form of discounting (this time by the probability of reward) that would cause avoidance of the more demanding tasks in exactly the same way.

Consider for example the N-back working memory task, which is frequently used in studies of cognitive effort (Westbrook et al., 2013). Higher levels of the N-back feel more effortful, but they also are intrinsically more difficult to perform accurately, because with more items to hold in memory, the maximum precision with which each item can be maintained is lower (Bays et al., 2009). If we observe discounting of the value of the task as the N-back level increases, it is impossible to say to what extent this is due to the greater effort required or the lower probability of completing a trial successfully and gaining reward. Similar arguments can be made for other effort manipulations, including visual attention (Apps et al., 2015), auditory attention (Crawford, Eisenstein, et al., 2022a; McLaughlin et al., 2021) and response conflict tasks (McGuire & Botvinick, 2010; Schmidt et al., 2012). This confounding may cause overestimation of participants’ cognitive effort sensitivity (because both effort and probability discounting are contributing to the observed behaviour). In order to dissociate these processes, it is essential that measures of cognitive effort hold difficulty constant when manipulating task demand.

It is important too that the difficulty of the task can be standardised across participants, as is usual in tasks manipulating physical effort (Chong et al., 2016; Husain & Roiser, 2018). For example, in physical effort tasks measuring grip strength (Bonnelle et al., 2016), the different levels are typically normalised relative to participants’ maximum grip force, so that while the absolute difficulty of the task (the grip force required) may differ between participants, the relative difficulty (the proportion of maximum capacity required) is held constant; crucially, therefore, participants are all able to achieve comparable levels of performance. Likewise, in cognitive effort tasks, we need to take account of baseline differences in cognitive capacity (e.g., differences in working memory capacity or processing speed). In tasks that do not standardise the difficulty, the problem of confounding again rears its head. If two participants apparently differ in the subjective value they ascribe to a task, we cannot say to what extent this reflects genuine differences in sensitivity to effort, as opposed simply to differences in the probability of achieving success. This is a particular concern with regards to psychiatric research into conditions such as depression and schizophrenia, where cognitive impairment is a core symptom (Gold et al., 2015; Mesholam-Gately et al., 2009; Rock et al., 2014) and therefore patients are likely to find the task fundamentally more difficult than healthy controls.

Some attempts have been made previously to take account of this issue. For example, Westbrook et al. (2013) statistically control for the effect of performance on subjective value in a regression model. However, this involves conditioning on a variable (performance), which is causally posterior to the outcome variable of the regression (subjective value). It is better to standardise the task itself, but to our knowledge this has been attempted only once before, by Ang et al. (2022), who devised a task they called the Cognitive Effort Motivation Task (CEMT). Ang et al. probed effort by asking participants to remember the location of one or more squares on a checkerboard and normalised the difficulty for each participant by reducing the total number of squares on the grid until they could achieve at least 80% accuracy during a training phase. However, although the CEMT has resolved the issue of confounding between participants, it still suffers from confounding *within* participants—as with the N-back task discussed above, higher demand levels in the CEMT give participants more information to hold in memory, such that both effort and the intrinsic difficulty of the task covary.

### A new cognitive effort measure—The Number Switching Task

The purpose of the present study was to develop a task that distinguishes cognitive effort discounting from probability discounting completely. Specifically, we targeted two main criteria: the manipulation of effort demand should not affect the probability of success; and it should be possible to standardise the task difficulty by reference to each participant’s baseline ability. While other tasks have been able to satisfy one or other of these criteria, the key significance of our task is that it meets both of them. Two further considerations were that the task should have several levels of effort demand, so that we could examine parametric responses to the manipulation across a reasonable dynamic range and also that it should be optimised for use online, where it is possible to obtain much larger sample sizes more practically than through in-person testing.

We developed the Number Switching Task (NST), which involves categorising each digit in a nine-digit sequence as either even or odd. By changing the frequency of switching between odd and even, we can manipulate the effort required but, crucially, it is only the order of the digits that changes—the actual content of the trials is all the same. This means that regardless of effort level, participants are completing the same set of operations on every trial, so the difficulty of the task—the information processing demands—is held constant (this distinguishes the NST from working memory based effort tasks, in which higher effort levels contain more information content to remember). Significantly, in the NST we can also standardise the difficulty of the task, by calibrating the time participants are allowed to complete each sequence, such that all participants are capable of achieving comparable levels of success.

Note that while this task is superficially similar to the Demand Selection Task (Kool et al., 2010), which also involves categorising digits, the manipulation of effort is rather different: the Demand Selection Task employs a Stroop-like structure of switching between different response rules, whereas in the NST it is the switching between odd and even itself that is effortful. This means that while the Demand Selection Task only compares two conditions, high vs. low switching, the NST is capable of measuring a wider range of effort levels (we used four effort levels). In addition the NST allows us to examine the interacting effect of reward on participants’ choices, unlike the Demand Selection Task which probes effort alone.

The primary purpose of this study was to validate the NST by testing the prediction that the effort manipulation will elicit the classic effort discounting effect without affecting the difficulty as measured by the rate of success. We also present some secondary analyses, including Bayesian modelling and an assessment of preliminary associations with cognitive traits relevant to depression and anhedonia.

## Methods

### Preregistration

This study was preregistered on the Open Science Framework (10.17605/OSF.IO/8Y7P9). There were no deviations from this plan.

### Participants

Participants were recruited through the online platform Prolific (www.prolific.co/). To be eligible, they had to be aged 18-60 years, with no history of a diagnosed mental health condition, and could not have taken part in any of the pilot experiments for this study. They also had to use a computer; smartphones or tablets were not allowed.

In our preregistration, we calculated a minimum required sample size of 259 participants to detect an effect of *r* = 0.2 with 90% power and alpha = 0.05 (two-tailed). We recruited more than this to allow for withdrawals and exclusions, so that the sample included 306 participants who completed the experiment. Of these, three were excluded, because they refreshed the web page part way through; nine were excluded, because they repeatedly failed the familiarisation phase of the effort task; and four were excluded, because they failed attention checks in the questionnaires. This left 290 participants with data included in the final analysis. The age and education distribution of this sample is provided in Table 1.

**Table 1:** Age and education distribution of participants

Age group (yr)	N
18-21	69
22-25	61
25-30	70
30-35	37
35-40	20
40-45	13
45-50	7
50-55	9
55-60	4
**Education**	**N**
Secondary education/high school	96
Higher apprenticeship	16
Foundation degree	7
Bachelor’s degree/Degree apprenticeship	106
Master’s degree	58
PhD	7

### Procedure

From Prolific, participants were automatically directed to another website, Gorilla (www.gorilla.sc/), where the study was hosted. There they completed the Number Switching Task, followed by eight questionnaires. At the end of the study, they were redirected back to Prolific via a unique URL, which allowed them to prove they had completed all the tasks; if instead they returned to Prolific manually (without this URL), their data were flagged and we checked whether they had actually completed all the tasks or not. On average, the entire study took approximately 45 minutes, from signing up to returning to Prolific, and participants were paid a flat rate of £5 plus a performance bonus of 1 pence per 3 points won on the effort task (on average participants won around £1.50 in bonuses).

#### Number Switching Task

**Fig. 1:**
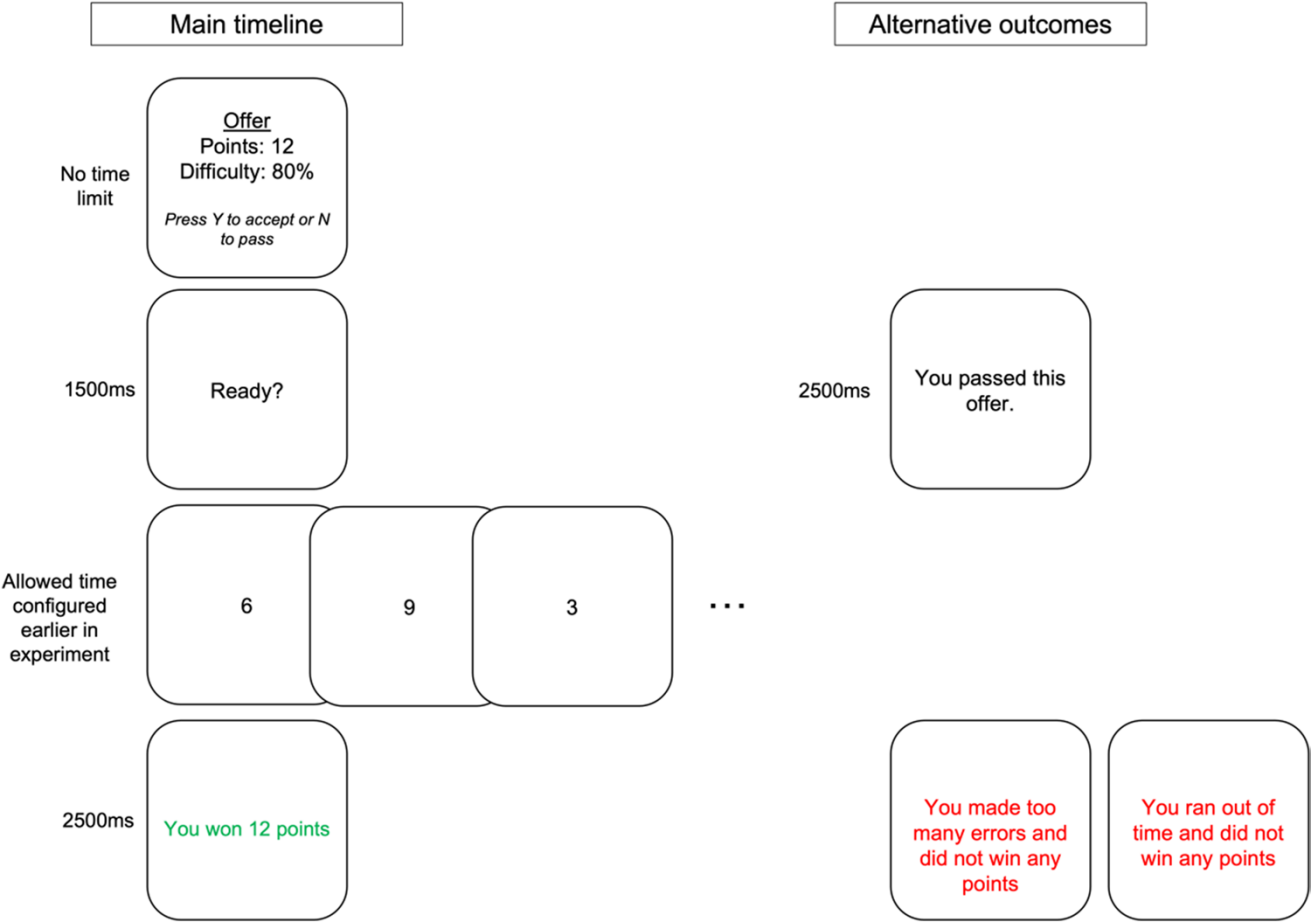
**Number Switching Task trial structure**. Participants chose whether to perform an effortful task depending on the points and effort level offered. If they accepted the offer, they were shown a random sequence of the digits 1-9 and had to indicate (by pressing the “f” or “j” keys) whether each of the digits was even or odd. Sequences with more frequent switching between odd and even were more effortful. To win the points on offer, participants had to categorise at least 8 of the 9 digits correctly and complete the sequence within the allowed time (which was calibrated to each individual). In the above figure, the “alternative outcomes” show screens that participants saw if they passed an offer or if they failed the trial because of too many errors or timing out

The structure of the task is shown in Fig. 1. On each trial, participants were offered a reward (3, 6, 9, or 12 points, corresponding to 1, 2, 3, or 4p of real money, respectively) to complete an effortful task with a specified level of demand. If they accepted this challenge, they had to complete the task successfully to win the reward; if they rejected it, they avoided performing the task but won no points and, after a timeout of 2,500 ms, proceeded to the next offer.

The effortful task itself was to categorise each of the digits in a random sequence of the numbers one to nine as either odd or even. The effort of this task scales with the frequency of switching between odd and even digits, allowing us to define four levels of demand: the lowest level, referred to in the task as 20%, contained either 1 or 2 switches; the next level (40%) 3 or 4 switches; the 60% level 5 or 6 switches; and the highest level, 80%, had 7 or 8 switches. On any given trial, the precise number of switches was determined at random to prevent the sequences becoming predictable. Participants were not explicitly told about the relationship between effort level and number of switches but instead learned this over the course of the familiarisation and main phases of the experiment.

As each digit appeared on the screen in sequence, participants responded, as soon as they saw the digits, using the “f” and “j” keys to indicate whether it was “odd” or “even” (key mapping was counterbalanced across participants). Instructions about these responses were provided on an instruction screen at the start of the task. While the individual categorisations were self-paced, meaning the next digit did not appear on the screen until a response had been made to the current item, there was a time limit for completing the overall sequence. A trial was marked as “correct” only if the sequence was finished within this limit and with no more than one wrong response. “Incorrect” sequences were not rewarded.

Importantly, this allowed us to standardise the difficulty across participants: by calibrating the allowed time based on performance during an earlier familiarisation phase, we ensured that all participants had similar success rates on the task.

##### Phases of the task

**Fig. 2:**
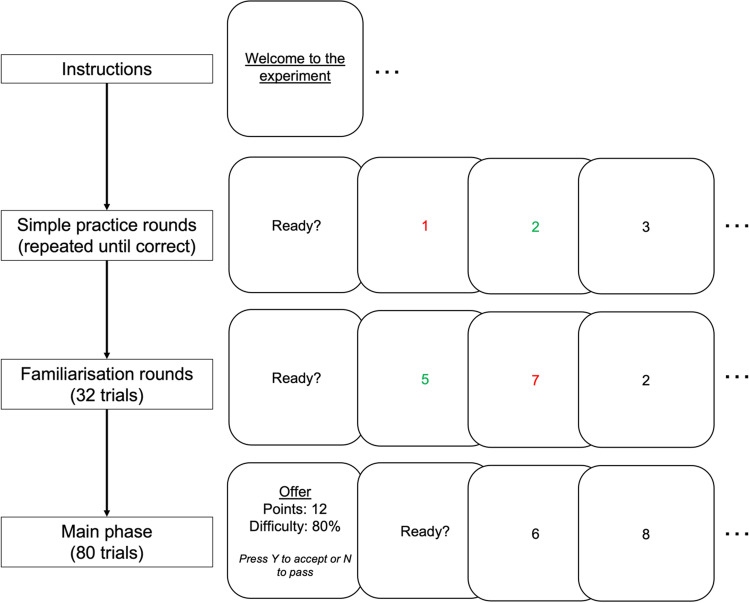
**Overall structure of the different phases of the task**. See main text for detailed description of each phase

Before embarking on the full task, participants progressed through instructions and practice rounds, followed by a longer familiarisation phase. This latter phase was used to calibrate the time limit for the sequences in the main phase of the task. See Fig. 2.

The instruction phase informed participants which keys they should press to categorise each digit as even or odd. Then, in an initial practice round, they were shown a sequence of the digits one to nine in ascending order, and they had to categorise all of these correctly to continue. If they made a single mistake, this round was repeated. We thus ensured that participants’ keys were working and that they understood the basic premise of the task. Unlike in the later, main phase of the task, here there was no time limit for completing the sequences. In addition, after participants gave their response the digits changed to green or red to indicate whether the response was correct or not (unlike in the main phase of the task where participants only received feedback at the end of the sequence).

The subsequent familiarisation phase was similar—participants completed a set of 32 sequences, again without the offer framing or the time limit—but now the digits were presented in a randomised order. This phase comprised four trials of each of the eight possible numbers of switches. Although there was no time limit, participants were now instructed to respond as quickly as possible while still trying to complete each sequence correctly.

To progress through the familiarisation phase to the main task, participants had to achieve at least 50% correct responses on the most difficult 8-switch trials; if they failed more than 50% of these trials they were given one opportunity to repeat this stage; if they failed again they were excluded.

For participants who passed the familiarisation phase, we calculated their time allowed for the main phase sequences as the median time to complete the hardest, 8-switch trials plus 500 ms. During piloting, we observed that this provided a good balance between providing sufficient time pressure to elicit the effort effects while ensuring that the task was possible within the maximum completion time for all participants.

Finally, participants completed the main phase of the task, in which the full trial structure was used (as depicted in Fig. 1). After 10 example trials in which participants were introduced to and gained experience with the offer framing, the main phase itself comprised a total of 80 trials: five trials of each of the 16 possible offer combinations, in a random order.

#### Questionnaire measures

Following the NST, participants completed a number of questionnaires. In all cases, participants gave their responses by moving a horizontal slider (which defaulted to the centre).

First was the NASA Task Load Index (Hart & Staveland, 1988), henceforth the “Subjective Task Load,” which asked participants to give their subjective rating (over six subscales) of the level of demand felt while performing each of the effort levels in the task. Subsequently, there were seven questionnaires assessing different traits: the Cognitive Complaints Inventory (Iverson & Lam, 2013), the Fatigue Severity Scale (Krupp et al., 1989), the International Physical Activity Questionnaire Short Form (Lee et al., 2011), the Need for Cognition six-item scale (Coelho et al., 2018), the Temporal Experience of Pleasure Scale (Gard et al., 2006) and the Zung Depression Scale (Zung, 1965). Further detail on these questionnaires and their scoring is provided in the Supplement.

We appended catch questions (e.g., “Select ‘very much’ for this question”) to two questionnaires—the Cognitive Complaints Inventory and the Need for Cognition Scale—to identify participants who were not paying attention. We placed these at the end of the questionnaires to avoid interfering with the psychometric properties of the measures themselves.

### Statistical analyses

#### Preregistered analyses

The main dependent variable on the NST was the proportion of offers accepted for each combination of reward and effort level. We also recorded participants’ accuracy and completion times for the odd/even categorisation task. These were of course conditional on participants accepting the offer in the first place and, in the case of the completion times, completing the sequence within the time allowed and with no more than one mistake allowed.

Our primary analysis was a multilevel (mixed effects) ANOVA. This was used because multilevel ANOVAs can accommodate unbalanced designs, which arise in this task because participants could choose to accept or reject trials at will, resulting in secondary measures (success rate and completion time) with different numbers of trials from each participant. These ANOVAs contained fixed effects of reward and effort and their interaction, and varying intercepts across subjects.

For analysis of the Subjective Task Load questionnaire, six multilevel ANOVAs were constructed, one for each of the constituent scales of the index, using a fixed effect of effort level and varying intercepts across subjects.

Throughout these analyses, we further investigated any significant effects indicated by the ANOVAs using post-hoc simple effects ANOVAs and paired-samples *t*-tests as appropriate. Note that, unlike the multilevel ANOVAs, the *t*-tests require complete cases. This results in differing degrees of freedom across analyses as some participants had to be excluded from specific post-hoc analyses of success rates or completion times if they had not completed any trials at a particular reward or effort level.

#### Exploratory analyses

##### Bayesian modelling

We considered 12 models, all variations on a logistic regression, listed in Table 2. The characteristic mathematical form of these models is provided in Equation 1. This particular set of equations represents Model 9; all of the other models can be constructed by modifying one or more components of this model.

**Table 2:** Specification of models fitted to the Number Switching Task. We fitted and compared twelve models in total, containing different terms. I = intercept, R = reward, E = effort. The prefix f or v indicates whether this term was fixed (one parameter estimated for the whole population) or varying (individual effects estimated for each participant, constrained by a hierarchical prior)

Model	
1	fI
2	fI + fR + fE
3	vI + fR + fE
4	vI + vR + vE
5	fI + fR + fE + f*E*^2^
6	vI + fR + fE + f*E*^2^
7	vI + vR + vE + v*E*^2^
8	vI + vR + v*E*^2^
9	vI + vR + v*R*^2^ + vE
10	vI + v*R*^2^+ vE
11	vI + vR + v*R*^2^ + vE + v*E*^2^
12	vI + v*R*^2^+ v*E*^2^

1 $${\displaystyle \begin{array}{c}{Y}_{subject, trial}\sim \textrm{Bernoulli}\left({p}_{subject, trial}\right)\\ {}{p}_{subject, trial}=\textrm{logistic}\Big({\alpha}_{subject}+{\beta}_{linear\_ reward, subject}{reward}_{trial}\\ {}+{\beta}_{quad\_ reward, subject}{reward_{trial}}^2+{\beta}_{effort, subject}{effort}_{trial}\Big)\end{array}}$$

where *y*_*subject*, *trial*_ ∈ {0, 1} is the choice of a particular subject on a particular trial to accept or reject the challenge. The underlying probability of accepting a challenge, *p*_*subject*, *trial*_, is then calculated as a logistic function of a linear combination of a number of parameters, typically including an intercept, *α*, and one or more effects of reward and effort, *β*_*reward*_ and *β*_*effort*_ respectively.

In Equation 1, the intercept and effects vary across subjects; however, as noted in Table 2, in some models these parameters were fixed instead, meaning all subjects took the same value.

The subject-level parameters were all given hierarchical priors, which were determined through a process of prior predictive checking. Details are given in the Supplement.

We standardised the values of the predictors (the reward and effort levels), for computational and arithmetical simplicity. Note that this affects the interpretation of absolute parameter values from the model.

The models were fitted using Markov Chain Monte Carlo simulation in Stan (Stan Development Team, 2021). Sampling was run for four chains each with 1,000 iterations. Subsequent to fitting, we performed the recommended standard diagnostics (visual inspection of the chains, no divergences or treedepth warnings, E-BFMI < 0.3, effective sample size > 400, split-$\hat{R}$ < 1.01; Betancourt, 2018) and found no issues. We also inspected the posterior predictions for the winning model and observed a good overall fit to the data (Fig. S7).

##### Structural equation modelling

We used confirmatory factor analysis (CFA) to fit several potential factor structures to the questionnaire data. We identified the best-fitting structure and inserted this into a structural equation model (SEM), with which we sought to predict the behavioural parameters estimated for each subject (intercept, reward, and effort sensitivity) from their cognitive trait scores.

#### Computing environment and packages

Analyses were conducted in R version 3.5.3 (R Core Team, 2019). We used the R package “lme4” (1.1-21; Bates et al., 2015) to fit the multilevel ANOVAs and “rstatix” (0.6.0; Kassambara, 2020) to conduct the post-hoc tests. Bayesian models were fitted in Stan using CmdStanR (0.3.0, Gabry and Češnovar, 2020). SEM was conducted in Lavaan (Rosseel, 2012).

## Results

### Preregistered analyses

#### Number switching task

##### Proportion of offers accepted

**Fig. 3:**
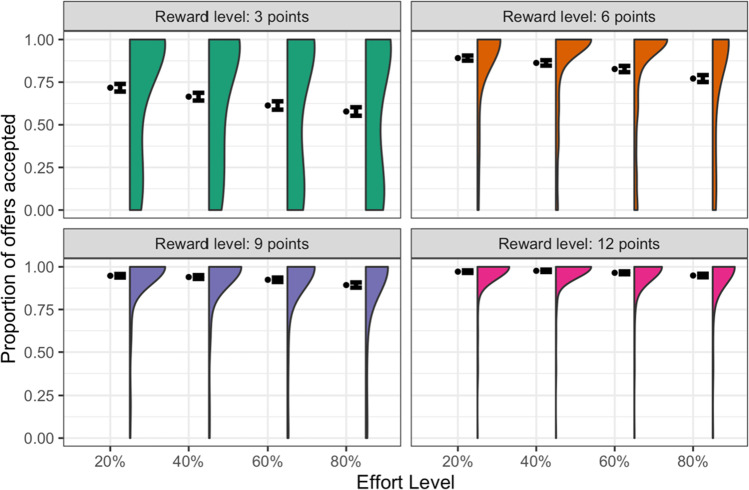
**Number Switching Task: proportion of offers accepted**. Mean, standard error, and distribution of the proportion of offers accepted for each combination of reward and effort level. See Fig. S9 for the same plot without faceting

The proportions of offers accepted at each level of reward and effort are plotted in Fig. 3. These show a significant reward-by-effort interaction, *F*(1, 4347) = 30.8, *p* < 0.001, η^2^_partial_ = 0.04, consistent with participants treating the effort level as an economic cost. Specifically, the value of a reward was progressively discounted as the effort required to obtain it increased, but this discounting was shallower when the reward offered was greater. Despite this flattening as reward increased, the effort effect was still significant at every reward level in post-hoc ANOVAs (all *p*s < 0.001). The main effects of reward and effort were also both significant, *F*(1,4347) = 108, *p* < 0.001, and *F*(1,4347) = 84.4, *p* < 0.001, respectively. Full descriptive statistics are provided in Table S1.

##### Success rate

**Fig. 4:**
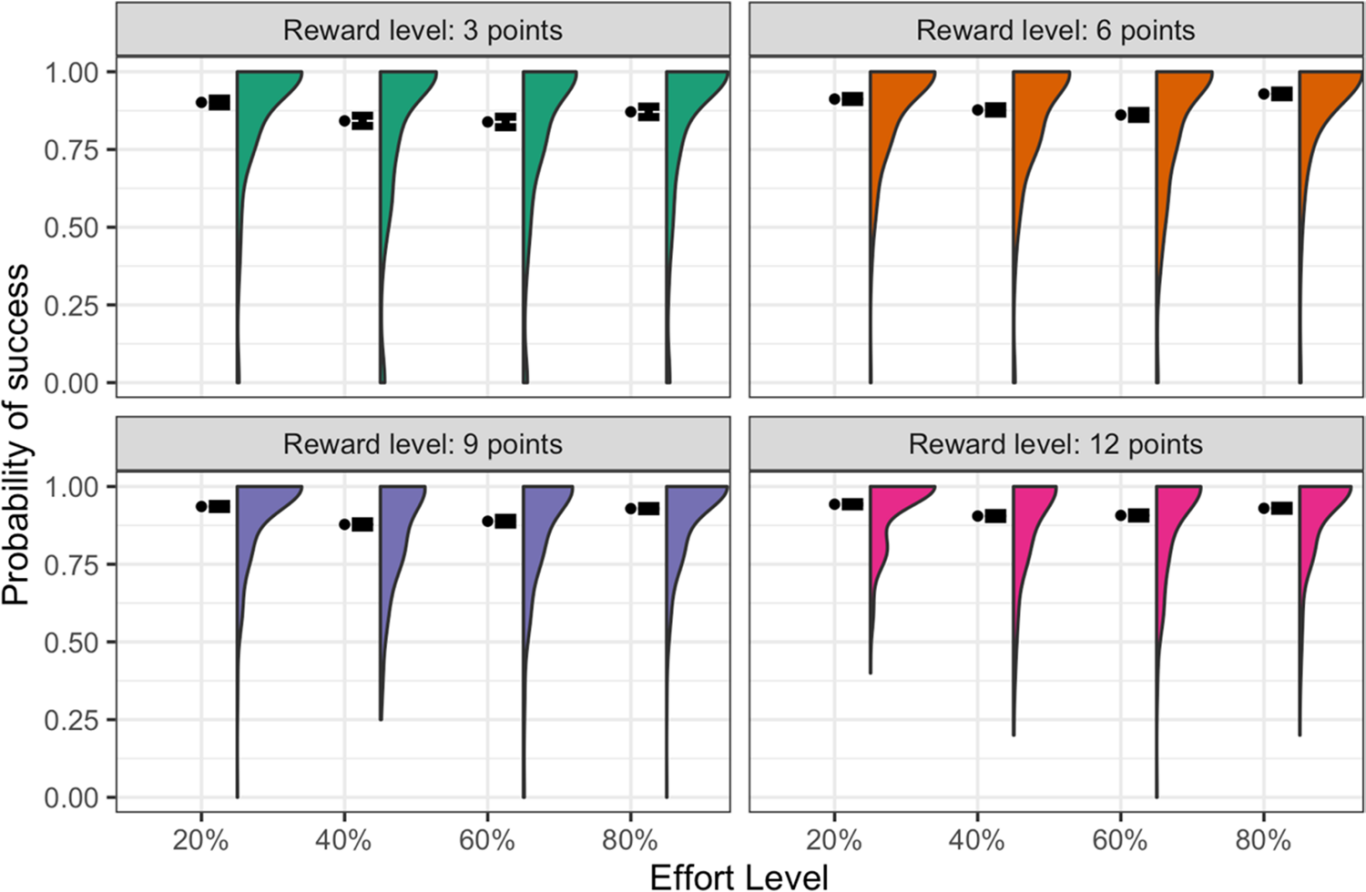
**Number Switching Task: proportion of trials completed successfully**. Mean, standard error, and distribution for each combination of reward and effort level. Trials were marked as “correct” if they were completed within the allowed time, with no more than one error. See Fig. S9 for the same plot without faceting

The success rate for each level of reward and effort is plotted in Fig. 4. The only statistically significant effect was that of reward, *F*(1, 4024) = 68.1, *p* < 0.0001, η^2^_partial_ = 0.08, with participants more likely to complete the sequence correctly as the offered reward increased (Table 3), consistent with higher rewards being more motivating. Post-hoc *t*-tests indicated that this was driven primarily by the increase in success rates between the 3- and 6-point reward levels, *t*(272) = 3.01, *p* = 0.008, *d* = 0.18, whereas the differences between 6 and 9 points, and 9 and 12 points did not achieve significance after Bonferroni-adjusting for multiple comparisons (*ps* = 0.10 and 0.07, and, *ds* = 0.13 and 0.14, respectively). Full descriptive statistics are provided in Table S2.

**Table 3:** Number Switching Task: Descriptive statistics for the proportion of trials completed successfully (across reward levels)

P(Success)		
Reward (points)	N	Mean (SD)
3	273	0.86 (0.18)
6	273	0.89 (0.12)
9	273	0.91 (0.10)
12	273	0.92 (0.10)

*Note*. To be marked as correct, sequences had to be completed within the time limit and with no more than one error. These data only include complete cases, i.e., where participants attempted at least one trial for each level of reward. *SD* standard deviation

The effort level had no significant effect on the success rate, *F*(1, 4024) = 2.18, *p* = 0.14, and the reward-by-effort interaction also was nonsignificant, *F*(1, 4024) = 0.380, *p* = 0.54.

Importantly, the success rate varied relatively little across participants (overall mean = 0.90, SD = 0.11), suggesting the standardisation of difficulty had been successful.

##### Completion times

**Fig. 5:**
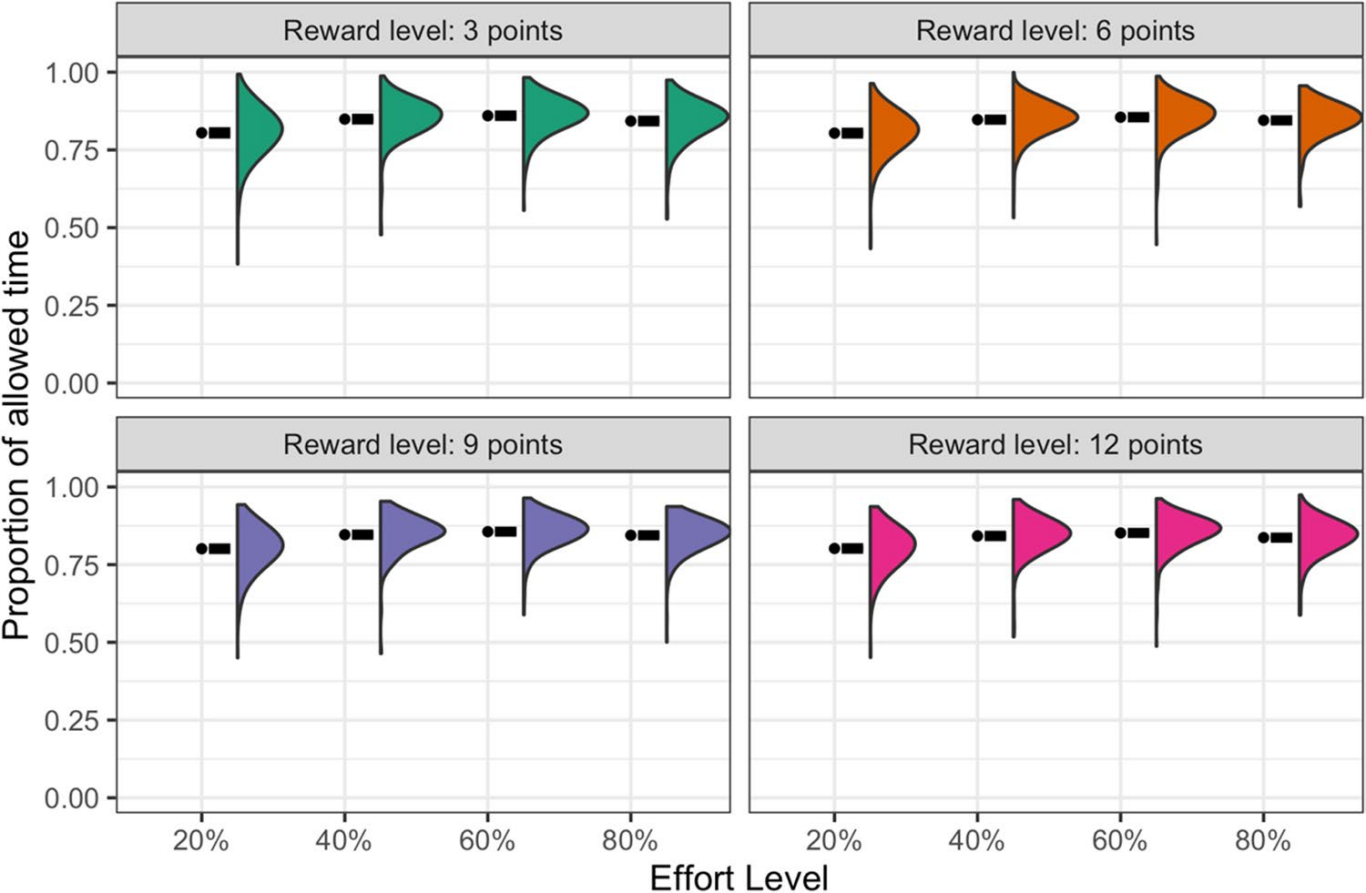
**Number Switching Task: completion time**. Mean, standard error, and distribution of the completion times (expressed as a proportion of each participant’s allowed time) for each level of reward and effort level. See Fig. S9 for the same plot without faceting

Completion times, expressed as a proportion of each participant’s maximum allowed time, are plotted in Fig. 5. Full descriptive statistics are provided in Table S3. The overall mean (SD) maximum allowed time was 8,953 ms (1,700 ms). There were significant main effects of both reward, *F*(1, 4014) = 10.1, *p* = 0.002, η^2^_partial_ = 0.03, and effort, *F*(1, 4014) = 610, *p* < 0.001, η^2^_partial_ = 0.52. The interaction effect was nonsignificant, *F*(1, 4014) = 0.56, *p* = 0.45. We further investigated the two main effects with three post-hoc *t*-tests for each factor. The *p*-values reported are Bonferroni-adjusted for multiple comparisons.

For the main effect of effort, we observed a curved pattern, with completion times lengthening progressively as the effort level increased between 20% and 60%, before decreasing again slightly for the 80% effort level (see descriptive statistics in Table 4). The contrasts between adjacent effort levels were all significant (20% vs. 40% effort: *t*(286) = 19.7, *p* < 0.001, *d* = 1.16; 40% vs. 60% effort: *t*(286) = 7.07, *p* < 0.001, *d* = 0.42; and 60% vs. 80% effort: *t*(286) = 8.88, *p* < 0.001, *d* = 0.52).

**Table 4:** Number Switching Task: Descriptive statistics for proportional completion time (across reward and effort levels)

Proportional completion time		
Reward (points)	N	Mean (SD)
3	273	0.84 (0.06)
6	273	0.84 (0.05)
9	273	0.84 (0.05)
12	273	0.83 (0.05)
Effort level		
20%	287	0.80 (0.07)
40%	287	0.85 (0.06)
60%	287	0.86 (0.05)
80%	287	0.84 (0.05)

*Notes.* Times are expressed as a proportion of each participant’s maximum allowed completion time. These data only include complete cases, i.e., where participants recorded at least one trial for each level of reward or points

For the main effect of reward, the descriptive statistics (Table 4) suggested that completion times decreased slightly with increasing reward level, although the post-hoc comparisons between adjacent reward levels were all non-significant following Bonferroni correction (3 vs. 6 points: *t*(272) = 0.08, *p* = 1.0; 6 vs. 9 points: *t*(272) = 1.11, *p* = 0.80; 9 vs. 12 points: *t*(272) = 2.32, *p* = 0.06).

Finally, in a further, exploratory analysis we also examined whether there was a significant association between participants’ maximum allowed time and the six questionnaire measures; however, none of these were significant (all *p*s > 0.05).

#### Subjective task load

**Fig. 6:**
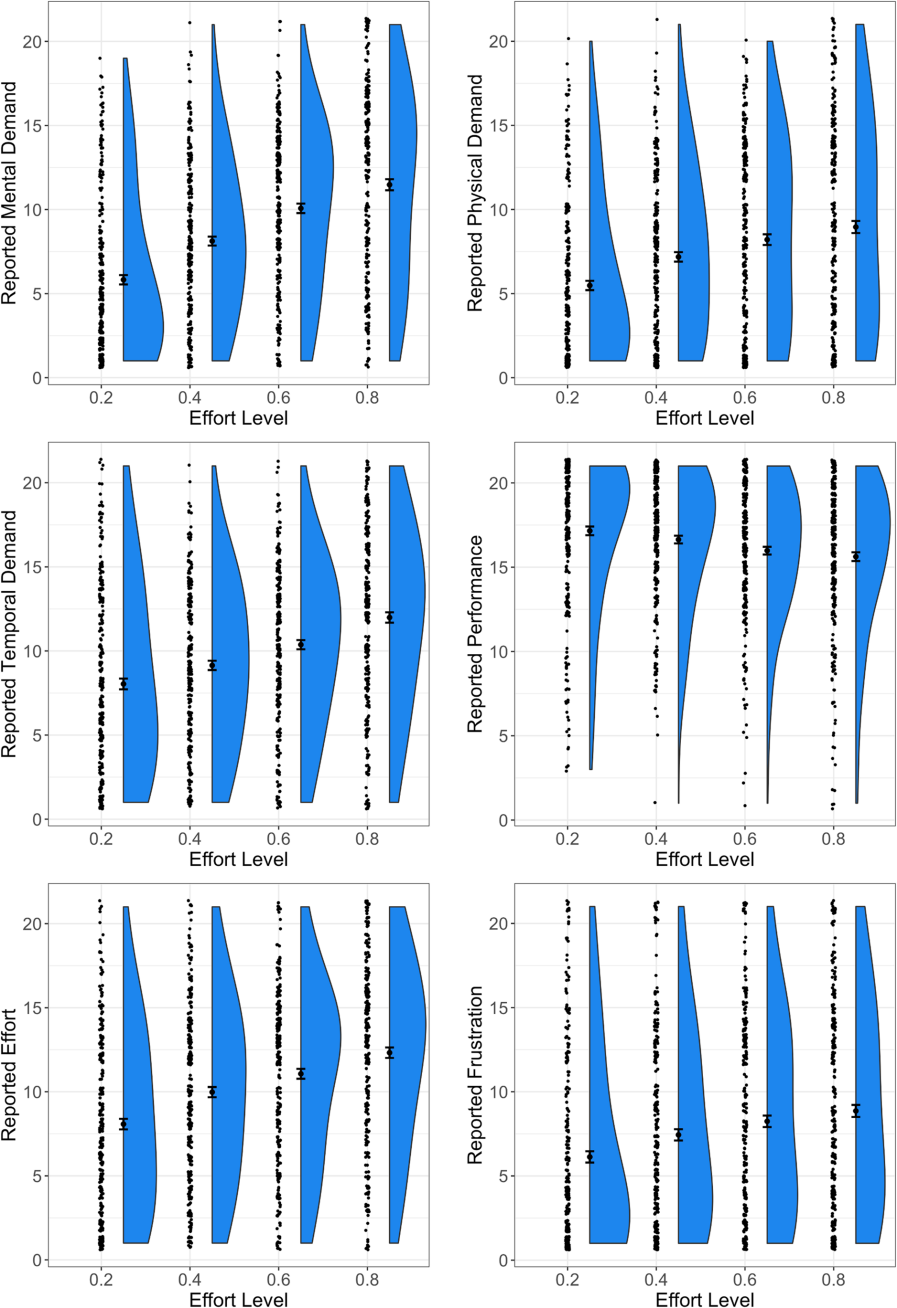
**Subjective task load ratings for each effort level***.* Plots show (from left to right within each plot) the individual data points, the means and standard errors, and the distributions of scores for each of the six scales of the index

Participants’ ratings of the subjective demand of each effort level are shown in Fig. 6, with each scale of the index plotted in a separate panel. Participants reported that they found each effort level successively more demanding, which was confirmed statistically (all ANOVAs indicated a significant effect of effort, *p*s < 0.0001). Post-hoc *t*-tests of the differences between sequential levels of effort are reported in Table 5. These comparisons were all significant (after Bonferroni correction), except for one: the comparison between ratings of perceived performance on the 60% and 80% effort.

**Table 5:** Subjective Task Load: post-hoc *t*-tests and standardised effect sizes

	Effort level comparisons								
	20% vs. 40%			40% vs. 60%			60% vs. 80%		
	*t*	*p*	*d*	*t*	*p*	*d*	*t*	*P*	*d*
Mental demand	10.2	<0.001	0.60	10.1	<0.001	0.59	5.65	<0.001	0.33
Physical demand	7.75	<0.001	0.46	5.45	<0.001	0.32	3.46	0.002	0.20
Temporal demand	4.50	<0.001	0.26	6.26	<0.001	0.37	7.15	<0.001	0.42
Performance	2.48	0.04	0.15	4.02	<0.001	0.24	1.96	0.15	0.12
Effort	8.38	<0.001	0.49	5.03	<0.001	0.30	5.52	<0.001	0.32
Frustration	6.13	<0.001	0.36	5.05	<0.001	0.30	2.93	0.01	0.17

Note. *P*-values above are corrected for three multiple comparisons within each scale of the index. Degrees of freedom are 289 throughout

### Exploratory analyses

#### Model comparison

We started by comparing the 12 models using the Widely-Applicable Information Criterion (WAIC; Watanabe, 2010). WAIC estimates the out-of-sample predictive accuracy of a model, providing both a point estimate and standard error, enabling us to quantify uncertainty.

**Fig. 7:**
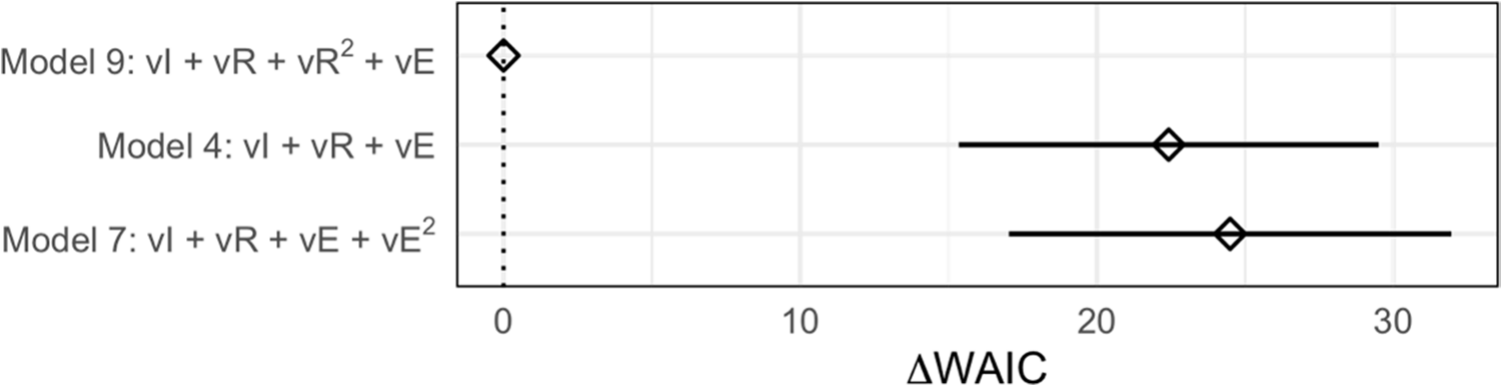
**Differences in WAIC relative to the best performing model (Model 9)**. Also shown is the standard error of this difference (black intervals). For simplicity, only the three best scoring models are shown above; the full plot of all eight models is given in Fig. S6. Model 9, the best performing model, contained a varying intercept, varying linear effects of reward and effort and a varying quadratic effect of reward

The WAIC values for the three best scoring models are plotted in Fig. 7. The best model was Model 9 (vI + vR + v*R*^2^ + vE), so we proceeded with the rest of the modelling analysis using the posterior estimates from this model. The distance to the next two models, Model 4 (vI + vR + vE) and Model 7 (vI + vR + vE + v*E*^2^), was fairly large but not definitive (3.2 and 3.3 standard errors respectively), so we also conducted sensitivity analyses using these two models, which are reported in the Supplement. There were no major differences between the inferences drawn from any of these models.

**Fig. 8:**
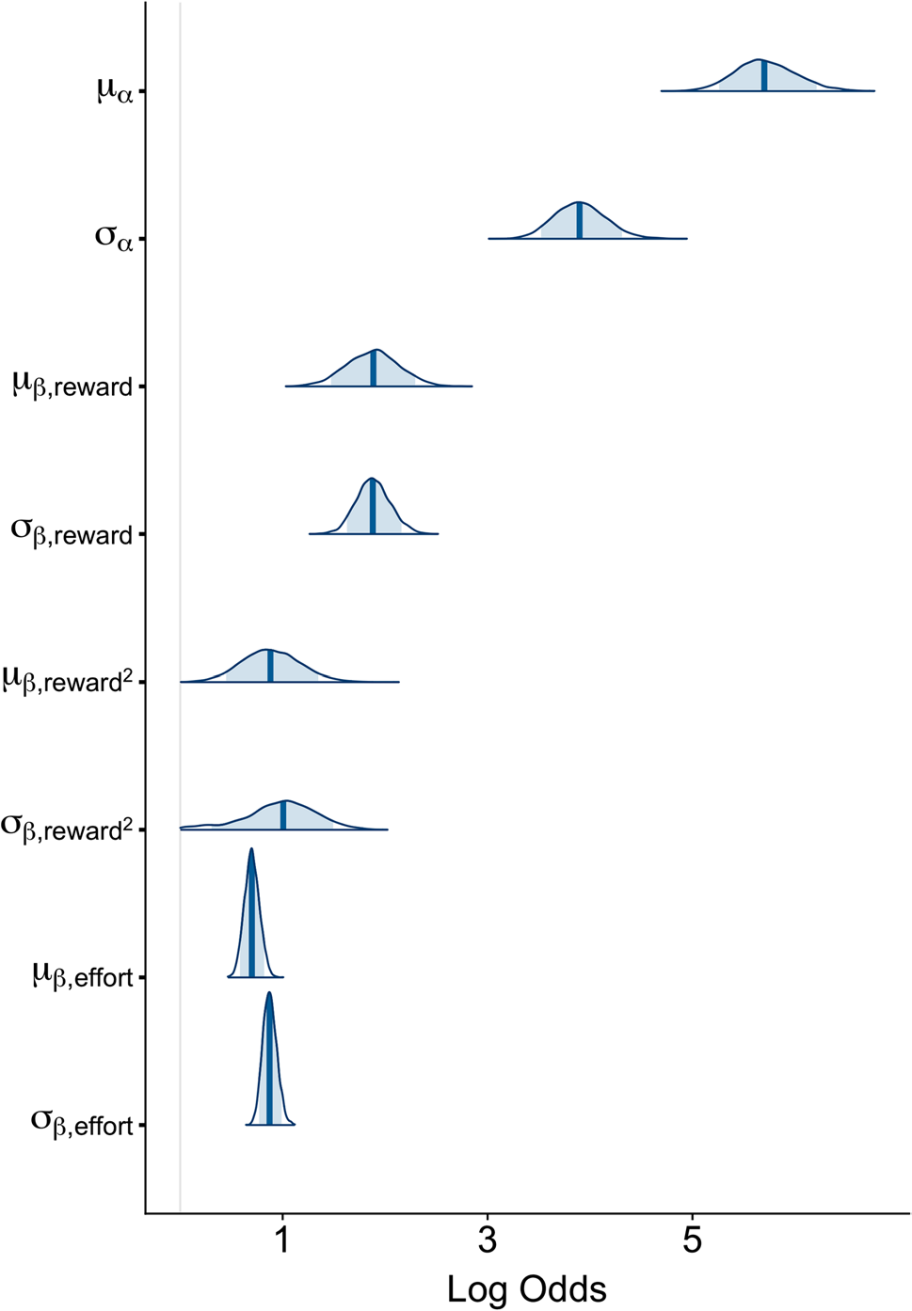
**Posterior distributions of the population level parameters from Model 9 (vI + vR + vR**^**2**^ **+ vE)**. The vertical line indicates the mean of each distribution and the shaded region the 89% quantile interval

Figure 8 shows the posterior estimates of the population-level intercept, reward and effort sensitivity parameters (mean and standard deviation). Note that none of these overlap zero, indicating that, as we would expect, participants are sensitive to both reward and effort (in accordance with the earlier ANOVA results).

The posterior predictions of the model are shown in Fig. S7. First, we see that the model predicts that the probability of accepting a challenge will decrease as a concave function of effort level and that this decline will be progressively shallower at higher levels of offered reward. This means that this model is able to reproduce not just the basic discounting of reward by effort, but also the specific shape of the discounting curves observed in the data. Second, the models also clearly show substantial uncertainty about the exact relationship between the probability of accepting a challenge and effort when it comes to predicting individual participant behaviour. In other words, the average population-level effect is clear but the model implies there is substantial variability between individuals.

**Fig. 9:**
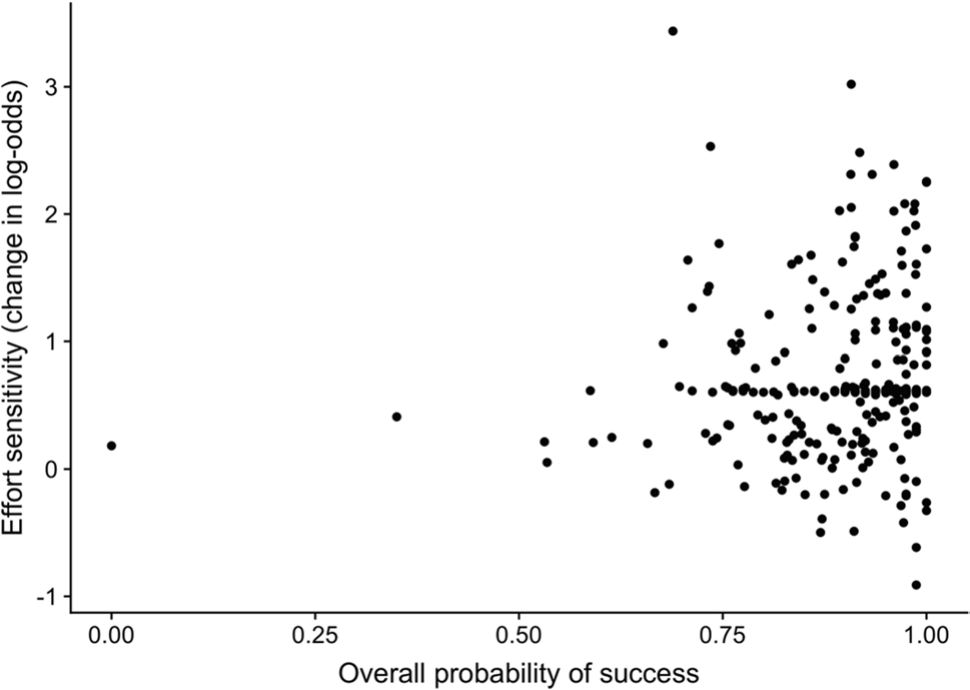
**Relationship between the probability of success and effort sensitivity**. The correlation was nonsignificant (*p* = 0.09), implying that effort sensitivity is not confounded by probability discounting in this task. Note the extreme point on the left of the graph corresponds to a participant who accepted (and failed) only one trial overall. In a sensitivity analysis, removing this participant increased the *p*-value of the correlation to *p* = 0.14

Next, we examined the correlation between the participant-level effort sensitivity parameters and the probability of success on the task (Fig. 9). Importantly, this was not significantly different from zero, *r*(288) = 0.10, *p* = 0.09, suggesting effort sensitivity was not confounded by probability discounting.

#### Association of computational parameters with questionnaire measures

The purpose of this final stage of our analysis was to explore any associations between the traits assessed by our questionnaires and the subject-level parameters estimated in Model 9 above (vI + vR + v*R*^2^ + vE).

As an exploratory step, we first conducted a series of regressions looking at each questionnaire individually (controlling for age and education each time). We found significant associations between effort sensitivity and Cognitive Complaints (*β* = 0.02, *p* = 0.04), Fatigue Symptoms (*β* = 0.01, *p* = 0.04), Need for Cognition (*β* = -0.02, *p* = 0.02), and Zung Depression Score (*β* = 0.01, *p* = 0.04). The remaining associations were nonsignificant: Experience of Pleasure (*p* = 0.90) and Physical Activity (*p* = 0.53). With regards to linear reward sensitivity we found an association with Need for Cognition (*β* = 0.04, *p* = 0.03), as well as with Physical Activity (*β* = −0.17, *p* = 0.03), but not the other questionnaire measures. Finally, there were no significant associations with any of the questionnaires for either quadratic reward sensitivity or the intercept parameter. Note, we have not corrected for multiple comparisons; we therefore emphasise that the above results should be treated as indicative and exploratory only.

To mitigate the multiple comparisons problem and provide a complementary means of assessing these associations, we had also planned a structural equation modelling analysis; this allowed us to test all of the associations at the same time, therefore without requiring correction. First, we used confirmatory factor analysis to compare several possible factor structures, which were devised *a priori*. The CFA is described in detail in the Supplement; briefly, the four structures considered were:

- One with a distinct latent factor for each questionnaire
- Another in which all questions mapped onto a single latent factor, equivalent to a ‘P’ factor in psychiatry (Caspi et al., 2013)
- A structure in which they were grouped by broad cognitive domain
- Another in which the questionnaires directly relevant to mental health symptoms were grouped together.

We found the “full” structure (i.e., with a distinct latent factor for each questionnaire) fitted the data best.

We then conducted an SEM, using this full factor structure as the measurement model, to predict the subject-level intercept, reward, and effort sensitivity parameters obtained from Model 9 (vI + vR + v*R*^2^ + vE) of the Number Switching Task. This time we found a significant positive association between Need for Cognition and both the linear and quadratic reward sensitivity parameters (standardised *β* = 0.29, *p* = 0.003, and *β* = 0.06, *p* = 0.03, respectively). However, all other associations were nonsignificant. A possible reason for the discrepancy between this result and those from the simple regressions detailed earlier is that the SEM regression measures the unique variance explained by each latent factor, whereas in fact several of the factors may be making a shared contribution to effort sensitivity. One clue that this is the case is that, in the SEM, the latent factors for cognitive complaints, fatigue, need for cognition, and depression symptoms all significantly covaried with one another. Full results from the SEM regression are provided in Tables S5 and S6.

#### Ruling out temporal discounting effects

One possible issue with the NST is that, because participants were allowed different times in which to complete the sequences, they may experience different rates of reward, in turn introducing temporal discounting effects. To test for this, we examined the association between participants’ probability of accepting each level and their average time taken to complete that level – if their choices were affected by temporal discounting we would expect to see a negative correlation, with lower probability of accepting an offer as the duration increases. However, there was no significant association, *r* = 0.002, *t*(4304) = 0.13, *p* = 0.89, suggesting temporal discounting did not affect participants’ choices. In a complementary analysis, we also tested the correlation between participants’ overall tendency to accept (the intercept parameter estimated by Model 9) and their allowed time; again this was nonsignificant, *r* = –0.03, *t*(0.53), *p* = 0.59. Further tests of the correlations between allowed time and other variables are included in the Supplement, Table S7.

## Discussion

We have presented a new task, the NST, for measuring cognitive effort and demonstrated that it resolves one of the major shortcomings of existing measures, namely the confounding of effort by task difficulty. In our results, obtained from a large online sample, participants treated higher effort levels as more costly, despite being just as likely to win the offered reward. The idea that cognitive effort is costly is one of the foundational assumptions of cognitive effort research and is supported by earlier studies showing effort discounting in several contexts (Apps et al., 2015; McLaughlin et al., 2021; Crawford, Eisenstein, et al., 2022a; Crawford, English, & Braver, 2022b; Kool et al., 2010; Kool & Botvinick, 2014; Westbrook et al., 2013); importantly, however, this is the first time that effort discounting has been studied in a task where effort and difficulty are not confounded. In other words, we were able to manipulate and measure cognitive effort without the problem of probability discounting.

A related concern was that we need to be able to standardise the difficulty of the task. Otherwise comparisons across participants (for example between patient groups and healthy controls) may not be valid. In the NST, this is achieved by tailoring the time allowed for completion of each sequence to participants individually. Encouragingly, the success rates were very consistent across participants, suggesting the standardisation procedure was successful.

The finding that completion times generally became longer as the effort level increased is consistent with these levels requiring more cognitive control (and therefore effort; Shenhav et al., 2017), but the small reduction in completion time at the highest level was unexpected. This is mirrored by a similar, although nonsignificant, increase in accuracy at the highest effort levels. The most likely explanation is that half of the sequences at this level involved alternating on every digit (i.e., eight switches), which, even though participants could not be sure exactly how many switches they would be shown, may have permitted them to respond slightly faster and/or more accurately. If so, this should be straightforward to address: future iterations of this task could use a ten- rather than nine-digit sequence, so that sequences that alternate on every digit are no longer possible. This would have the further advantage of requiring one digit to be shown twice, making it impossible for participants to work out which digits remain to be shown.

A key component of the NST’s design is that the time allowed to complete the nine-digit sequences is personalised to each participant. This allows us to normalise the difficulty of the task and adjust for differences between participants in cognitive capacity. However, it also introduces the potential for temporal discounting, because participants may experience different rates of reward. Fortunately, we were able to rule out effects of temporal discounting in our results; we found that there was no association between participants’ effort sensitivity and the time they had to complete the sequences. Thus, we can be relatively confident that the NST is measuring effort discounting rather than temporal discounting.

One last feature of the NST results that deserves comment is the observation that the acceptance rates are relatively high (averaging >50%) across all of the conditions. This is by design – the task needed to be suitable for use with patients, who are likely to have much lower acceptance rates than the healthy participants we have initially tested here. Nevertheless, if in future studies the acceptance rates are seen to be too high, then a possible change might be, for example, to offer a low reward/low effort alternative during the choice phase, rather than the current accept/reject choice. This also could permit calculation of subjective indifference points if desired.

The Subjective Task Load results show clearly that participants reported finding each level of effort progressively more demanding. The increase in physical demand at higher effort levels is a potential issue, as participants should in theory be making the same number of key presses at approximately the same rate across all levels. This result may be genuine—for example, the time pressure on the task could be felt as a kind of physical demand, causing participants to tense up—but it also could suggest the presence of experimenter demand effects. One way to address this in the future would be to modify the language used in the offers; rather than referring to difficulty levels, it may be preferable for the offers to refer to the switching rate explicitly instead (alternatively, abstract shapes or colours could be used to label each effort level, avoiding words entirely). That said, we also found that in post-hoc tests the only nonsignificant difference was on the performance subscale, between the two highest effort levels, which directly matches the behavioural result discussed above, that participants performed slightly faster at 80% than 60% effort. That participants were sensitive to this detail gives some reassurance that the Subjective Task Load results were accurate appraisals after all.

The remainder of our analyses were exploratory in nature and aimed principally at demonstrating how this task can be used for individual differences research. The most parsimonious model according to WAIC included linear and quadratic effects of reward and a linear effect of effort, all of which varied across participants. However, we should be clear that this model is linear on a log-odds scale only, implying nonlinear (specifically convex) effort costs on the outcome scale, consistent with other work (Ritz et al., 2021). The model also indicated there was substantial variability in effort sensitivity across participants, which will be beneficial for individual differences research.

In a planned analysis, we used SEM to measure the association between several trait measures and the participant-level parameters from the behavioural model. The only significant association was between Need for Cognition, a construct representing participants’ enjoyment of cognitively demanding activity, and reward sensitivity, the extent to which participants’ choices changed in line with the offered reward. Possibly this is because participants who score higher on Need for Cognition pay more attention to the parameters of the task. While the lack of other associations, particularly with effort sensitivity, was somewhat surprising, one issue may be overlap between the multiple questionnaire measures that we included. Ideally this would have been fully accounted for by the factor analysis. Nevertheless, we saw remaining covariance between the latent factors in the SEM. As we mention in the results section above, this covariance may mean that the factors are making a shared contribution to effort sensitivity, which is not detected by the SEM (because the latter only measures the unique contribution of each factor).

In line with this explanation, when we examined the relationship between each questionnaire and the outcome variables in separate regressions, we found that there were indeed significant associations between effort sensitivity and a number of the mental health-related questionnaires (Cognitive Complaints Inventory, Fatigue Severity Scale, and Zung Depression Scale). In addition the negative association with Need for Cognition, meaning that participants who enjoy cognitive demanding activity were less sensitive to effort, provides particular reassurance about the external validity of the model. We nevertheless urge caution about not overinterpreting the results of the simple regressions alone; with many tests performed we unavoidably run into the problem of multiple comparisons, so these results should only be regarded as exploratory for now.

Finally, we are optimistic about the use of the NST in clinical populations, but again more validation will clearly be needed to support this. In addition, further work could be conducted to design more sophisticated models. Those presented in this paper represent a starting point, and natural extensions would be explicitly to model the correlations between the sensitivity parameters, or add a lapse component that acknowledges that on some trials participants may simply decide at random. Numerous other model variations can be devised and built and would be interesting topics of study.

## Conclusions

We have presented a new task measuring cognitive effort, which resolves a longstanding problem of conflating the effort demanded by a task with its difficulty. Not only have we been able to manipulate effort without changing the difficulty of the task, but we can additionally standardise the difficulty across participants by tailoring the time allowed according to performance at an individual level. This is the first cognitive effort task in which such standardisation can be achieved and it means that individual differences research can be performed without concerns around confounding from difficulty or ability.

## Supplementary information

ESM 1 (PDF 1.99 MB)

## Data Availability

This study was preregistered on the Open Science Framework, 10.17605/OSF.IO/8Y7P9. All data and analysis scripts are provided at the Open Science Foundation repository, 10.17605/OSF.IO/X34KN, and code to run the Number Switching Task is deposited in the Gorilla Open Materials Repository, https://app.gorilla.sc/openmaterials/328049.
