# Correction: Measuring cognitive effort without difficulty

**DOI:** 10.3758/s13415-023-01078-4

**Published:** 2023-02-23

**Authors:** Hugo Fleming, Oliver J. Robinson, Jonathan P. Roiser

**Affiliations:** 1 Institute of Cognitive Neuroscience, University College London, 17 Queen Square, London, WC1N 3AZ UK; 2 MRC Cognition and Brain Sciences Unit, University of Cambridge, 15 Chaucer Road, Cambridge, CB2 7EF UK

**Correction: Cognitive, Afective, & Behavioral Neuroscience**

**https://doi.org/10.3758/s13415-023-01065-9**

The original article has been updated to include middle initial ‘P’ in co-author’s name Jonathan P. Roiser.

**Open Access**

This article is licensed under a Creative Commons Attribution 4.0 International License, which permits use, sharing, adaptation, distribution and reproduction in any medium or format, as long as you give appropriate credit to the original author(s) and the source, provide a link to the Creative Commons license, and indicate if changes were made. The images or other third party material in this article are included in the article's Creative Commons licence, unless indicated otherwise in a credit line to the material. If material is not included in the article's Creative Commons licence and your intended use is not permitted by statutory regulation or exceeds the permitted use, you will need to obtain permission directly from the copyright holder. To view a copy of this license, visit http://creativecommons.org/licenses/by/4.0/.

