# Supplementary material for: Measuring cognitive effort without difficulty
Source: Cogn Affect Behav Neurosci. 2023 Feb 7;23(2):290–305. doi: 10.3758/s13415-023-01065-9 (PMC10050044; doi:10.3758/s13415-023-01065-9)
Supplement: Supplementary file 1 — (PDF 1.99 MB) [file 13415_2023_1065_MOESM1_ESM.pdf]

Supplementary Materials

Method

Questionnaires

NASA Task Load Index. The NASA Task Load Index (Hart & Staveland, 1988), referred to in the main text as the 'Subjective Task Load', assesses subjective workload on six subscales: mental demand, physical demand, temporal demand, performance, effort and frustration. For each subscale and, in our case, for each level of effort, participants were asked to rate their experience of the task on a 21-point scale from 'very low' to 'very high'. The six subscales were presented in the same order as above, within which the questions about the different effort levels were randomised. We report participants' scores on each subscale for each effort level separately.

Cognitive Complaints Inventory. The Cognitive Complaints Inventory (Iverson & Lam, 2013) is a six-item questionnaire in which participants rate their problems with concentration, memory and thinking skills, on a four-point scale from 0 (not at all) to 3 (very much). We report total scores (where higher scores indicate more cognitive complaints).

We appended a catch question ("Select 'very much' for this question") to this questionnaire to identify participants who were not paying attention. We placed this at the end to avoid interfering with the psychometric properties of the questionnaire itself.

Fatigue Severity Scale. The Fatigue Severity Scale (Krupp, LaRocca, Muir-Nash & Steinberg, 1989) is a nine-item questionnaire in which participants rate their experience of

fatigue and the impact fatigue has on their daily activities, on a scale from 1 to 7. We report total scores (where higher scores indicate more fatigue).

International Physical Activity Questionnaire Short Form. The International Physical Activity Questionnaire Short Form (IPAQ-SF; Lee, Macfarlane, Lam & Stewart, 2011) is a seven-item scale that measures self-assessed physical activity over the previous seven days. Participants are asked on how many days and on average for how long each day they spent engaged in vigorous activity, moderate activity, walking and sitting. These estimates are weighted by their estimated metabolic requirements and summed to generate an overall score (termed 'MET-minutes', a way of expressing activity relative to a resting metabolic rate), as follows:

- If necessary, bouts of activity are truncated at a maximum of three hours.
- Walking MET-minutes per week = 3.3 * walking minutes * walking days
- Moderate MET-minutes per week = 4 * walking minutes * walking days
- Vigorous MET-minutes per week = 8 * walking minutes * walking days
- Total MET-minutes per week = Walking Met-minutes + Moderate Met-minutes + Vigorous Met-minutes

Need for Cognition Scale (6-item version). The six-item Need for Cognition Scale (Coelho, Hanel & Wolf, 2018) measures the extent to which participants enjoy engaging in difficult cognitive activity. Participants rate each of six statements from 1 (not characteristic of themselves) to 5 (characteristic). We report participants' total scores (where higher scores indicate greater enjoyment of cognitively demanding activity).

We added another catch question to the end of this questionnaire.

Temporal Experience of Pleasure Scale. The Temporal Experience of Pleasure Scale (TEPS; Gard, Gard, Kling & John, 2006) is an 18-item scale with two subscales: a 10-item anticipatory pleasure scale and an 8-item consummatory scale. Each item consists of a statement (e.g. "The smell of freshly cut grass is enjoyable to me") which participants rate on a 6-point scale from 'very false for me' to 'very true for me'. We report total scores (where higher scores indicate greater disposition to experience of pleasure or, equivalently, lower anhedonia).

Zung Depression Scale. The Zung Depression Scale (Zung, 1965) is a 20-item questionnaire in which participants respond to a series of statements about how they might feel on a 4-point scale from 'a little of the time' to 'most of the time'. We report total scores (where higher scores indicate more depressive symptoms).

Computational Models

Full specification of models

Model 1. Varying Intercept

Equation S1

$$Y_{subject,trial} \sim \text{Bernoulli}(p_{subject})$$

$$p_{subject} = \text{logistic}(\alpha_{subject})$$

$$\alpha_{subject} \sim Normal(\mu_\alpha, \sigma_\alpha)$$

$$\mu_\alpha \sim Normal(0, 1.5)$$

$$\sigma_\alpha \sim Exponential(2)$$

Model 2. Fixed Intercept and Fixed Linear Effects of Reward and Effort

Equation S2

$$Y_{subject,trial} \sim \text{Bernoulli}(p_{trial})$$

$$p_{trial} = \text{logistic}(\alpha + \beta_{reward} reward_{trial} + \beta_{effort} effort_{trial})$$

$$\alpha \sim Normal(0, 1.5)$$

$$\beta_{reward} \sim Normal(0, 1)$$

$$\beta_{effort} \sim Normal(0, 1)$$

Model 3. Varying Intercept and Fixed Linear Effects of Reward and Effort

Equation S3

$$Y_{subject,trial} \sim \text{Bernoulli}(p_{subject,trial})$$

$$p_{subject,trial} = \text{logistic}(\alpha_{subject} + \beta_{reward} reward_{trial} + \beta_{effort} effort_{trial})$$

$$\alpha_{subject} \sim Normal(\mu_\alpha, \sigma_\alpha)$$

$$\mu_\alpha \sim Normal(0, 1.5)$$

$$\sigma_\alpha \sim Exponential(2)$$

$$\beta_{reward} \sim Normal(0, 1)$$

$$\beta_{effort} \sim Normal(0, 1)$$

Model 4. Varying Intercept and Varying Linear Effects of Reward and Effort

Equation S4

$$Y_{subject,trial} \sim \text{Bernoulli}(p_{subject,trial})$$

$$p_{subject,trial} = \text{logistic}(\alpha_{subject} + \beta_{reward,subject} reward_{trial} + \beta_{effort,subject} effort_{trial})$$

$$\alpha_{subject} \sim Normal(\mu_\alpha, \sigma_\alpha)$$

$$\mu_\alpha \sim Normal(0, 1.5)$$

$$\sigma_{\alpha} \sim Exponential(2)$$

$$\beta_{reward,subject} \sim Normal(\mu_{reward}, \sigma_{reward})$$

$$\mu_{reward} \sim Normal(0,1)$$

$$\sigma_{reward} \sim Exponential(2)$$

$$\beta_{effort,subject} \sim Normal(\mu_{effort}, \sigma_{effort})$$

$$\mu_{effort} \sim Normal(0,1)$$

$$\sigma_{effort} \sim Exponential(2)$$

Model 5. Fixed Intercept, Fixed Linear Effect of Reward and Fixed Linear and Quadratic Effects of Effort

Equation S5

$$Y_{subject,trial} \sim \text{Bernoulli}(p_{trial})$$

$$p_{trial} = \text{logistic}(\alpha + \beta_{reward} reward_{trial} + \beta_{effort} effort_{trial}$$

$$+ \beta_{quad\_effort} effort_{trial}{}^{2})$$

$$\alpha \sim Normal(0, 1.5)$$

$$\beta_{reward} \sim Normal(0, 1)$$

$$\beta_{effort} \sim Normal(0, 1)$$

$$\beta_{quad\_effort} \sim Normal(0, 1)$$

Model 6. Varying Intercept, Fixed Linear Effect of Reward and Fixed Linear and Quadratic Effects of Effort

Equation S6

$$Y_{subject,trial} \sim \text{Bernoulli}(p_{subject,trial})$$

$$p_{subject,trial} = \text{logistic}(\alpha_{subject} + \beta_{reward} reward_{trial} + \beta_{effort} effort_{trial} + \beta_{quad\_effort} {effort_{trial}}^2)$$

$$\alpha_{subject} \sim Normal(\mu_\alpha, \sigma_\alpha)$$

$$\mu_\alpha \sim Normal(0, 1.5)$$

$$\sigma_\alpha \sim Exponential(2)$$

$$\beta_{reward} \sim Normal(0, 1)$$

$$\beta_{effort} \sim Normal(0, 1)$$

$$\beta_{quad\_effort} \sim Normal(0, 1)$$

Model 7. Varying Intercept, Varying Linear Effect of Reward and Varying Linear and Quadratic Effects of Effort

Equation S7

$$Y_{subject,trial} \sim \text{Bernoulli}(p_{subject,trial})$$

$$p_{subject,trial} = \text{logistic}(\alpha_{subject} + \beta_{reward,subject} reward_{trial} + \beta_{effort,subject} effort_{trial} + \beta_{quad\_effort,subject} {effort_{trial}}^2)$$

$$\alpha_{subject} \sim Normal(\mu_\alpha, \sigma_\alpha)$$

$$\mu_\alpha \sim Normal(0, 1.5)$$

$$\sigma_\alpha \sim Exponential(2)$$

$$\beta_{reward,subject} \sim Normal(\mu_{reward}, \sigma_{reward})$$

$$\mu_{reward} \sim Normal(0,1)$$

$$\sigma_{reward} \sim Exponential(2)$$

$$\beta_{effort,subject} \sim Normal(\mu_{effort}, \sigma_{effort})$$

$$\mu_{effort} \sim Normal(0,1)$$

$$\sigma_{effort} \sim Exponential(2)$$

$$\beta_{quad\_effort,subject} \sim Normal(\mu_{quad\_effort}, \sigma_{quad\_effort})$$

$$\mu_{quad\_effort} \sim Normal(0,1)$$

$$\sigma_{quad\_effort} \sim Exponential(2)$$

<u>Model 8.</u> Varying Intercept, Varying Linear Effect of Reward and Varying Quadratic Effect of Effort

Equation S8

$$Y_{subject,trial} \sim \text{Bernoulli}(p_{subject,trial})$$

$$p_{subject,trial} = \text{logistic}(\alpha_{subject} + \beta_{reward,subject} reward_{trial}$$

$$+ \beta_{quad\_effort,subject} effort_{trial}{}^{2})$$

$$\alpha_{subject} \sim Normal(\mu_{\alpha}, \sigma_{\alpha})$$

$$\mu_{\alpha} \sim Normal(0, 1.5)$$

$$\sigma_{\alpha} \sim Exponential(2)$$

$$\beta_{reward,subject} \sim Normal(\mu_{reward}, \sigma_{reward})$$

$$\mu_{reward} \sim Normal(0,1)$$

$$\sigma_{reward} \sim Exponential(2)$$

$$\beta_{effort^2,subject} \sim Normal(\mu_{effort^2}, \sigma_{effort^2})$$

$$\mu_{effort^2} \sim Normal(0,1)$$

$$\sigma_{effort^2} \sim Exponential(2)$$

Model 9. Varying Intercept, Varying Linear and Quadratic Effects of Reward and Varying Linear Effect of Effort

Equation S9

$$Y_{subject,trial} \sim \text{Bernoulli}(p_{subject,trial})$$

$$p_{subject,trial} = \text{logistic}(\alpha_{subject} + \beta_{reward,subject} reward_{trial}$$
$$+ \beta_{quad\_reward,subject} {reward_{trial}}^2 + \beta_{effort,subject} effort_{trial})$$

$$\alpha_{subject} \sim Normal(\mu_\alpha, \sigma_\alpha)$$

$$\mu_\alpha \sim Normal(0, 1.5)$$

$$\sigma_\alpha \sim Exponential(2)$$

$$\beta_{reward,subject} \sim Normal(\mu_{reward}, \sigma_{reward})$$

$$\mu_{reward} \sim Normal(0,1)$$

$$\sigma_{reward} \sim Exponential(2)$$

$$\beta_{quad\_reward,subject} \sim Normal(\mu_{quad\_reward}, \sigma_{quad\_reward})$$

$$\mu_{quad\_reward} \sim Normal(0,1)$$

$$\sigma_{quad\_reward} \sim Exponential(2)$$

$$\beta_{effort,subject} \sim Normal(\mu_{effort}, \sigma_{effort})$$

$$\mu_{effort} \sim Normal(0,1)$$

$$\sigma_{effort} \sim Exponential(2)$$

Model 10. Varying Intercept, Varying Quadratic Effect of Reward and Varying Linear Effect of Effort

Equation S10

$$Y_{subject,trial} \sim \text{Bernoulli}(p_{subject,trial})$$

$$p_{subject,trial} = \text{logistic}(\alpha_{subject} + \beta_{quad\_reward,subject} {reward_{trial}}^2$$
$$+ \beta_{effort,subject} effort_{trial})$$

$$\alpha_{subject} \sim Normal(\mu_\alpha, \sigma_\alpha)$$

$$\mu_\alpha \sim Normal(0, 1.5)$$

$$\sigma_\alpha \sim Exponential(2)$$

$$\beta_{quad\_reward,subject} \sim Normal(\mu_{quad\_reward}, \sigma_{quad\_reward})$$

$$\mu_{quad\_reward} \sim Normal(0,1)$$

$$\sigma_{quad\_reward} \sim Exponential(2)$$

$$\beta_{effort,subject} \sim Normal(\mu_{effort}, \sigma_{effort})$$

$$\mu_{effort} \sim Normal(0,1)$$

$$\sigma_{effort} \sim Exponential(2)$$

<u>Model 11.</u> Varying Intercept, Varying Linear Effects of Reward and Effort and Varying Quadratic Effects of Reward and Effort

Equation S11

$$Y_{subject,trial} \sim \text{Bernoulli}(p_{subject,trial})$$

$$p_{subject,trial} = \text{logistic}(\alpha_{subject} + \beta_{reward,subject} reward_{trial} + \beta_{effort,subject} effort_{trial} + \beta_{quad\_reward,subject} {reward_{trial}}^2 + \beta_{quad\_effort,subject} {effort_{trial}}^2)$$

$$\alpha_{subject} \sim Normal(\mu_\alpha, \sigma_\alpha)$$

$$\mu_\alpha \sim Normal(0, 1.5)$$

$$\sigma_\alpha \sim Exponential(2)$$

$$\beta_{reward,subject} \sim Normal(\mu_{reward}, \sigma_{reward})$$

$$\mu_{reward} \sim Normal(0,1)$$

$$\sigma_{reward} \sim Exponential(2)$$

$$\beta_{effort,subject} \sim Normal(\mu_{effort}, \sigma_{effort})$$

$$\mu_{effort} \sim Normal(0,1)$$

$$\sigma_{effort} \sim Exponential(2)$$

$$\beta_{quad\_reward,subject} \sim Normal(\mu_{quad\_reward}, \sigma_{quad\_reward})$$

$$\mu_{quad\_reward} \sim Normal(0,1)$$

$$\sigma_{quad\_reward} \sim Exponential(2)$$

$$\beta_{quad\_effort,subject} \sim Normal(\mu_{quad\_effort}, \sigma_{quad\_effort})$$

$$\mu_{quad\_effort} \sim Normal(0,1)$$

$$\sigma_{quad\_effort} \sim Exponential(2)$$

Model 12. Varying Intercept, Varying Quadratic Effects of Reward and Effort

Equation S12

$$Y_{subject,trial} \sim \text{Bernoulli}(p_{subject,trial})$$

$$p_{subject,trial} = \text{logistic}(\alpha_{subject} + \beta_{quad\_reward,subject} reward_{trial}{}^{2}$$

$$+ \beta_{quad\_effort,subject} effort_{trial}{}^{2})$$

$$\alpha_{subject} \sim Normal(\mu_{\alpha}, \sigma_{\alpha})$$

$$\mu_{\alpha} \sim Normal(0, 1.5)$$

$$\sigma_{\alpha} \sim Exponential(2)$$

$$\beta_{quad\_reward,subject} \sim Normal(\mu_{quad\_reward}, \sigma_{quad\_reward})$$

$$\mu_{quad\_reward} \sim Normal(0,1)$$

$$\sigma_{quad\_reward} \sim Exponential(2)$$

$$\beta_{quad\_effort,subject} \sim Normal(\mu_{effort^2}, \sigma_{effort^2})$$

$$\mu_{quad\_effort} \sim Normal(0,1)$$

$$\sigma_{quad\_effort} \sim Exponential(2)$$

Prior Predictive Checks

Below are plotted the distributions of all of the parameters used in the eight models, which are expressed and plotted on the logodds scale. Additionally we include plots of the prior predictive distributions for the probability of accepting an offer for an individual subject, $p_{subject}$. These are on the probability scale. Where the plots show simulated, rather than analytical, distributions, these represent 1000 simulations with 100 hypothetical participants in each. In all cases, the shaded distributions are 66%, 95% and 100% quantiles.

Priors for the intercepts:

Shown below are the prior distributions for the two types of intercept parameters, fixed (Figure S1) and varying (Figure S2) intercepts. In both cases we see that the priors chosen represent conservative predictions about the data we would expect to observe. Most importantly, a participant's probability of accepting an offer, $p_{subject}$, is constrained to be between 0 and 1. Within that range however the prior density is distributed fairly uniformly, save that it drops off below about 0.2 and above about 0.8. Overall these priors encode beliefs about participants' average acceptance rates that slightly downweight the likelihood of observing the most extreme values but otherwise are fairly agnostic.

Figure S1. Prior predictions for the fixed intercept parameter (featured in models 1 and 5), with distribution $\alpha \sim Normal(0,\ 1.5)$.
(a) shows the analytical distribution of the intercept α itself, whilst (b) shows the implied prior predictions for the probability of accepting an offer for an individual subject.

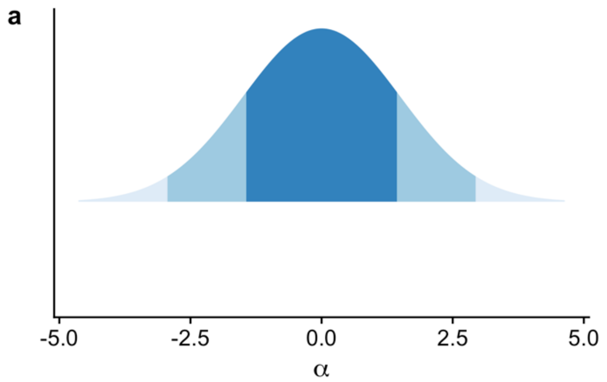

Figure S2. Prior predictions for the varying intercept parameters (featured in models 2, 3, 4, 6, 7, 8, 9, 10, 11 and 12), with distributions $\mu_\alpha \sim Normal(0, 1.5)$, $\sigma_\alpha \sim Exponential(2)$ and $\alpha_{subject} \sim Normal(\mu_\alpha, \sigma_\alpha)$. (a) and (b) show the analytical distributions of, respectively, the population level average intercept and the standard deviation of this average; (c) shows the distribution of the (subject level) intercepts themselves; and (d) shows the implied prior predictions for the probability of accepting an offer for an individual subject.

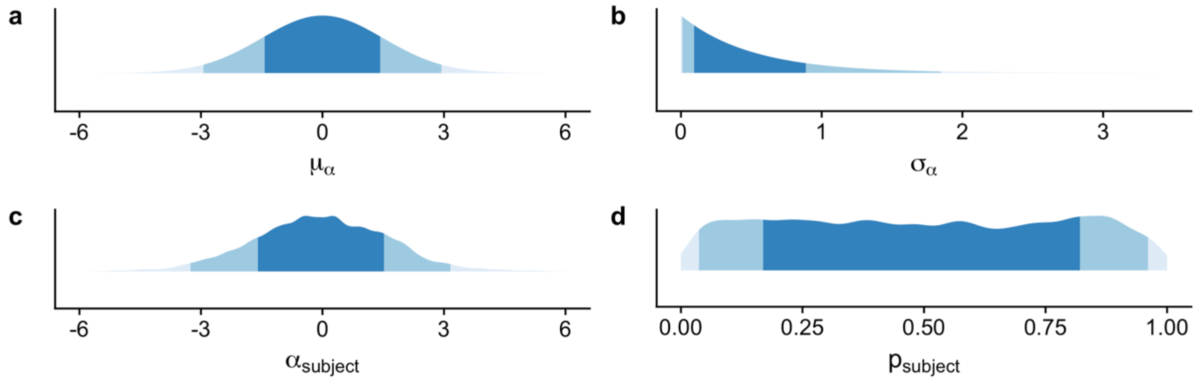

Priors for the reward/effort effects

Shown below are the prior distributions for the reward/effort sensitivity parameters, for the fixed linear (Figure S3) and varying linear (Figure S4) cases and for the fixed and varying quadratic effort sensitivity parameter (Figure S5).

The most important plot for interpreting these priors is in the bottom right of each box, labelled b (or d in the case of Figure S4). This shows the prior on the effect of the reward/effort manipulation on the probability scale. Specifically, this is the predicted change in a participant's probability of accepting the offer when one of the manipulations is changed by one level. For example, moving from 3 to 6 points (or 6 to 9 points, etc.), while effort is kept constant, or vice versa moving from 80% to 60% effort (or 60% to 40% effort, etc.) while reward is kept constant.

In all cases the priors chosen encode conservative beliefs that the effects, if present, are expected to be approximately in the range 0 – 0.25, within which, because of the rightward skew, smaller effects are considered more likely than larger ones.

Figure S3. Prior predictions for the fixed linear reward and effort sensitivity parameters (featured in models 1, 3, 5 and 6), with distribution $\beta \sim Normal(0,\ 1)$.
(a) shows the analytical distribution of the sensitivity β itself, whilst (b) shows the implied predictions for the change in probability of accepting an offer as the reward or effort changes by one level (e.g. from 3 to 6 points, or 80% to 60% effort etc.)

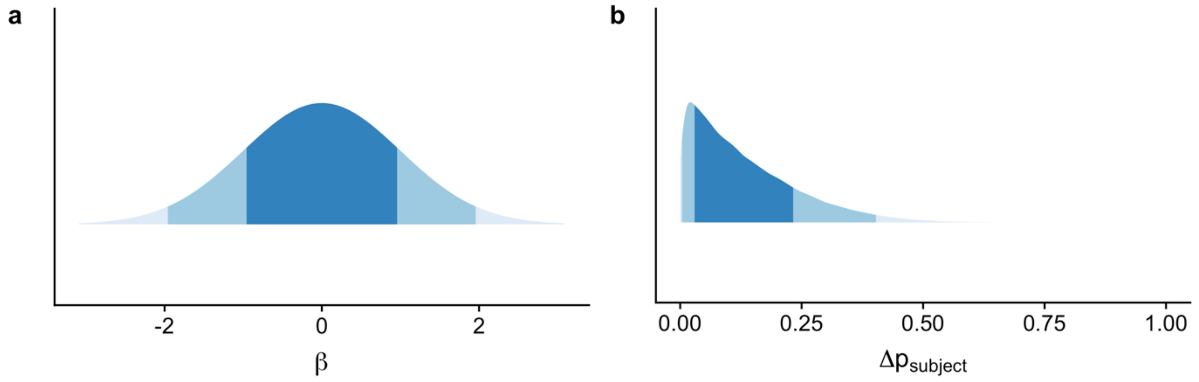

Figure S4. Prior predictions for the varying linear reward and effort sensitivity parameters (featured in models 4, 7, 8, 9, 10 and 11), with distributions $\mu_\beta \sim Normal(0,1)$, $\sigma_\beta \sim Exponential(2)$ and $\beta_{subject} \sim Normal(\mu_\beta\,, \sigma_\beta)$.
(a) and (b) show the analytical distributions of, respectively, the population level average sensitivity and the standard deviation of this average; (c) shows the distribution of the (subject level) sensitivity parameters themselves; and (d) shows the implied predictions for the change in probability of accepting an offer as the reward or effort changes by one level (e.g. from 3 to 6 points, or 80% to 60% effort etc.)

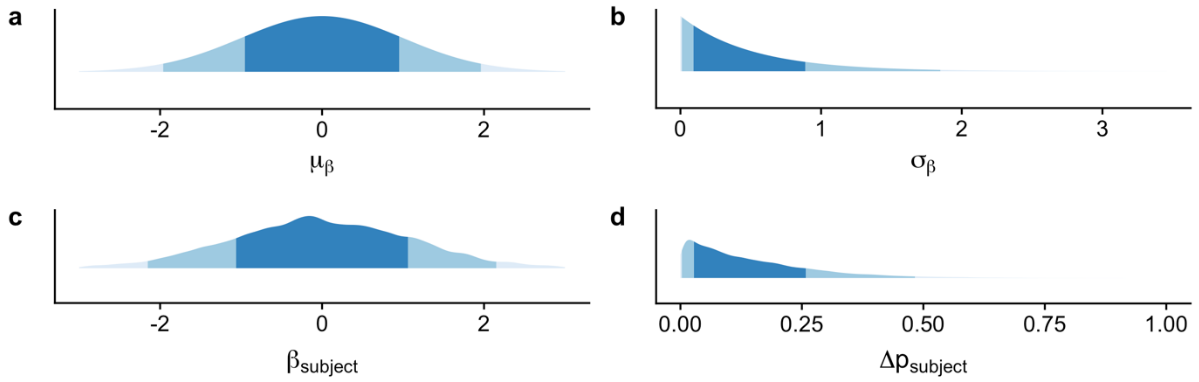

Figure S5. Prior predictions for the quadratic reward/effort sensitivity parameters with distributions $\beta_{quad\_effort} \sim Normal(0, 1)$ (fixed effect in models 5 and 6) and $\mu_{quad\_effort} \sim Normal(0,1)$, $\sigma_{quad\_effort} \sim Exponential(2)$ and $\beta_{quad\_effort,subject} \sim Normal(\mu_{quad\_effort}\,, \sigma_{quad\_effort})$ (varying effects in models 7, 8, 9, 10, 11 and 12). The distributions of the sensitivity parameters themselves are the same as for the linear parameters plotted above, in Figure S3(a) for the fixed effect, and in Figure S4(a, b and c) for the varying effects. Below we plot the implied predictions for the change in probability of accepting an offer as the required effort changes by one level (e.g. from 80% to 60% effort etc.), for (a) a fixed parameter, and (b) varying

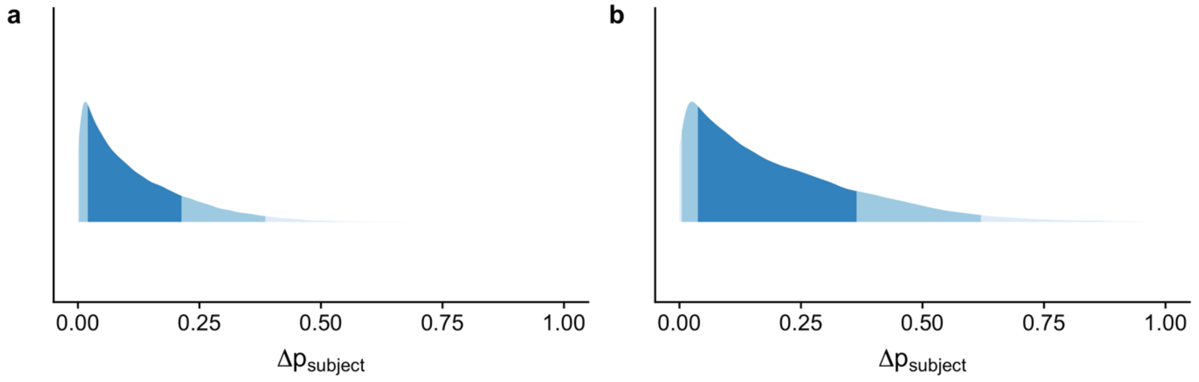

Supplementary Results

Full descriptive statistics for the Cognitive Effort Task

Table S1. Cognitive Effort Task: Proportion of Trials Accepted.		
	**P(accept)**	
**Reward (points)**	**N**	**Mean (SD)**
3	290	0.64 (0.37)
6	290	0.84 (0.27)
9	290	0.93 (0.19)
12	290	0.97 (0.12)
**Effort level**		
20%	290	0.88 (0.18)
40%	290	0.86 (0.19)
60%	290	0.83 (0.21)
80%	290	0.80 (0.25)
**Reward: Effort**		
3: 20%	290	0.72 (0.38)
3: 40%	290	0.66 (0.39)
3: 60%	290	0.61 (0.42)
3: 80%	290	0.58 (0.43)
6: 20%	290	0.89 (0.25)
6: 40%	290	0.86 (0.27)

6: 60%	290	0.83 (0.31)
6: 80%	290	0.77 (0.35)
9: 20%	290	0.95 (0.17)
9: 40%	290	0.94 (0.19)
9: 60%	290	0.92 (0.22)
9: 80%	290	0.89 (0.26)
12: 20%	290	0.97 (0.13)
12: 40%	290	0.98 (0.11)
12: 60%	290	0.96 (0.14)
12: 80%	290	0.95 (0.17)

Table S2. Cognitive Effort Task: Proportion of Trials Completed Successfully.

	P(success)	
**Reward (points)**	**N**	**Mean (SD)**
3	273	0.86 (0.18)
6	287	0.89 (0.13)
9	289	0.91 (0.10)
12	289	0.92 (0.10)
**Effort level**		
20%	289	0.92 (0.10)
40%	288	0.88 (0.12)
60%	289	0.88 (0.15)
80%	287	0.92 (0.12)
**Reward: Effort**		
3: 20%	255	0.90 (0.20)
3: 40%	247	0.84 (0.26)
3: 60%	227	0.84 (0.25)
3: 80%	218	0.87 (0.24)
6: 20%	280	0.91 (0.16)
6: 40%	278	0.88 (0.19)
6: 60%	271	0.86 (0.21)
6: 80%	263	0.93 (0.17)

9: 20%	287	0.94 (0.13)
9: 40%	285	0.88 (0.17)
9: 60%	281	0.89 (0.18)
9: 80%	277	0.93 (0.14)
12: 20%	288	0.94 (0.11)
12: 40%	288	0.90 (0.16)
12: 60%	287	0.91 (0.17)
12: 80%	285	0.93 (0.13)

Table S3. Cognitive Effort Task: Completion time.		
**Proportional completion time**		
**Reward (points)**	**N**	**Mean (SD)**
3	273	0.84 (0.06)
6	286	0.84 (0.05)
9	289	0.84 (0.05)
12	289	0.83 (0.05)
**Effort level**		
20%	289	0.80 (0.07)
40%	288	0.85 (0.06)
60%	288	0.86 (0.05)
80%	287	0.84 (0.05)
**Reward: Effort**		
3: 20%	255	0.80 (0.08)
3: 40%	247	0.85 (0.07)
3: 60%	225	0.86 (0.06)
3: 80%	216	0.84 (0.06)
6: 20%	280	0.80 (0.07)
6: 40%	278	0.85 (0.06)
6: 60%	268	0.86 (0.06)

6: 80%	263	0.85 (0.06)
9: 20%	286	0.80 (0.07)
9: 40%	285	0.85 (0.06)
9: 60%	278	0.86 (0.05)
9: 80%	277	0.84 (0.05)
12: 20%	288	0.80 (0.07)
12: 40%	288	0.84 (0.06)
12: 60%	287	0.85 (0.06)
12: 80%	285	0.84 (0.06)

Bayesian Modelling

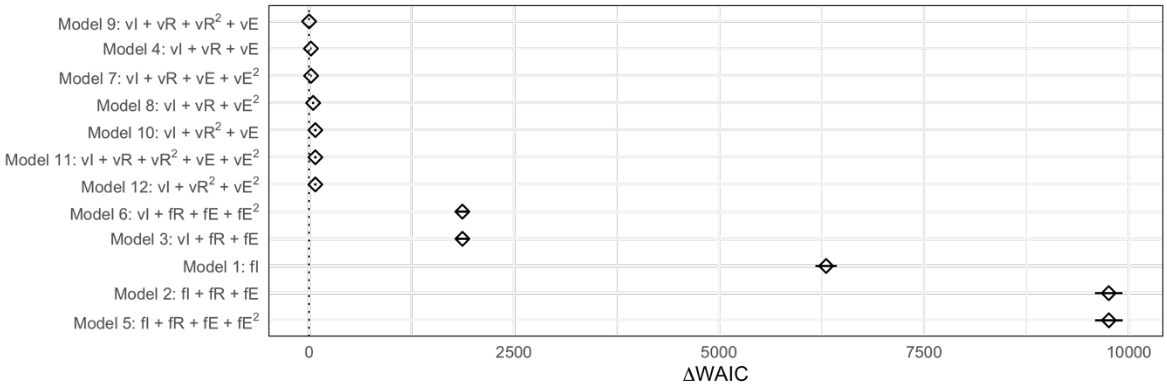

Figure S6. *Differences in WAIC relative to the best performing model (Model 4)*. Full plot of all eight models that were considered. This figure is a counterpart to Figure 6 in the main text.

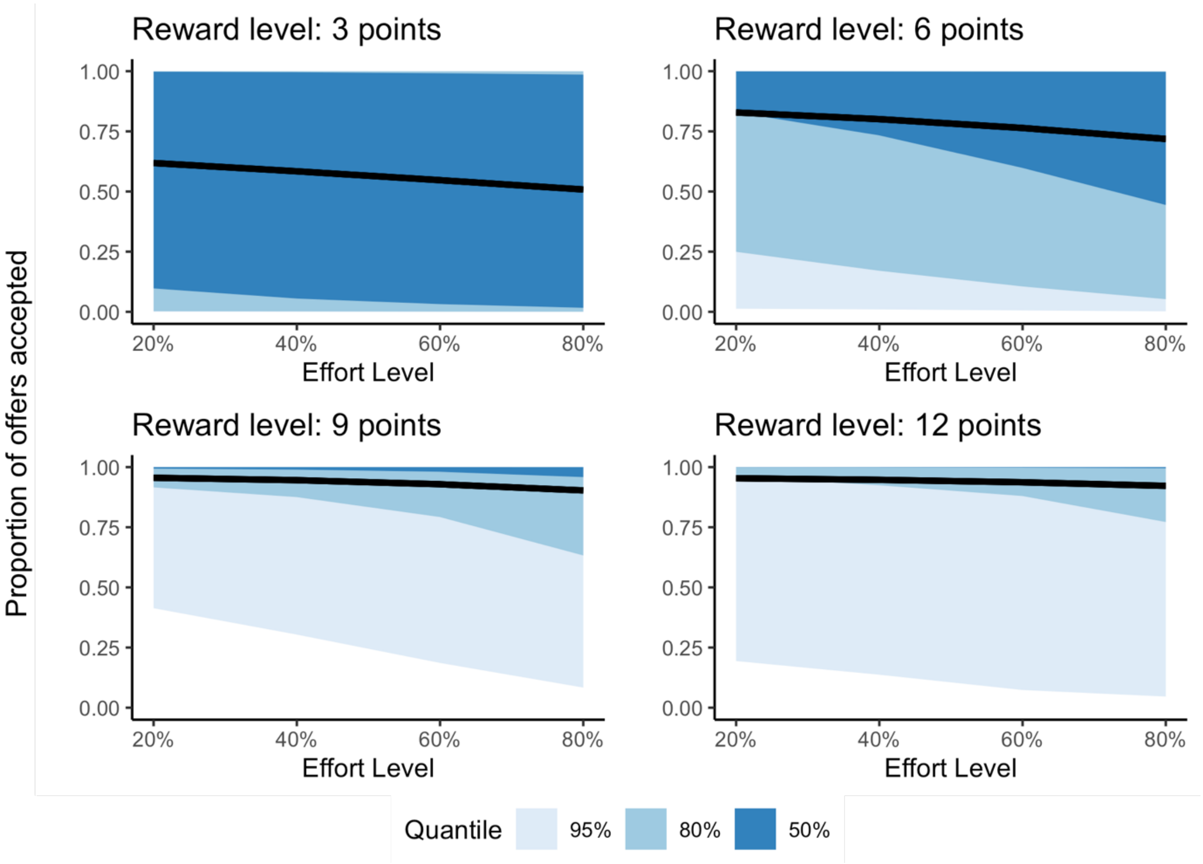

Figure S7. *Posterior predictions for Model 9* (vI + vR + vR^2 + vE). Plots show mean (black line) and posterior quantiles (95%, 80% and 50%) of the predicted probability of accepting an offer across each level of reward and effort. Note that these are predictions for simulated new participants and therefore incorporate uncertainty not just about the average effect of the manipulations in the population, but also about the behaviour of individual participants.

Structural Equation Modelling

We considered four factor structures:

- One with a distinct latent factor for each questionnaire
- Another in which the all questions mapped onto a single latent factor, equivalent to a ‘P’ factor in psychiatry (Caspi et al., 2013)
- A structure in which they were grouped by broad cognitive domain
- Another in which the questionnaires directly relevant to mental health symptoms were grouped together

These are shown graphically in Figure S8 (a) – (d) below.

We ran confirmatory factor analyses with each of these models and then compared their relative model fits using three metrics – overall log likelihood, Akaike’s Information Criterion and the Bayesian Information Criterion. The results are presented in the Table S4 below. The ‘Full’ structure, with a distinct latent factor for each questionnaire, consistently fitted the data best across all three measures.

Table S4. Results of model comparison for the confirmatory factor analysis.

**Factor Structure**	**Log Likelihood**	**AIC**	**BIC**
Full structure	-27237	54756	55273
MH symptoms grouped	-27741	55740	56213
Cognitive domain	-27827	55912	56386
‘P’-like structure	-28281	56814	57277

Using this winning factor structure, we then constructed a structural equation model to predict our three subject-level parameters from the computational model using the latent factors for the questionnaires, plus the demographics measures (age and level of education). The results are presented in Table S5 below.

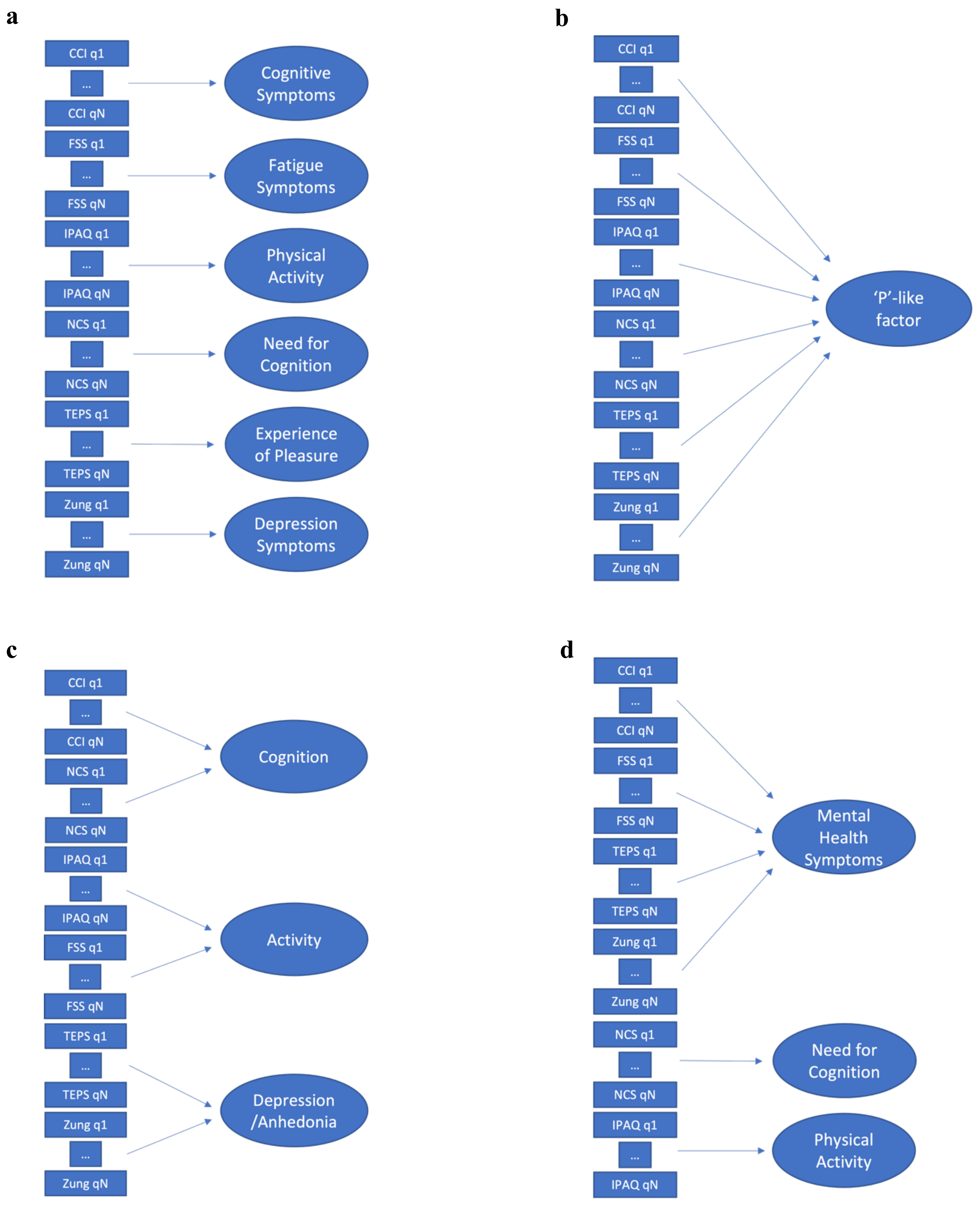

Figure S8. *The four factor structures compared in the confirmatory factor analysis.* (a) A full factor structure, with a distinct latent factor for each questionnaire. (b) A minimal factor structure, with just a single factor onto which all the questions loaded, corresponding to a 'p'-like factor. (c) An intermediate structure in which questionnaires were grouped by broad cognitive domain. (d) Another intermediate structure, in which the questionnaires directly relevant to mental health symptoms were grouped together.

Table S5. Results of the structural equation model: regression coefficients and test results.

**Path**	**Standardised Coefficient**	***z*-score**	***p***
Intercept →			
- Age	0.09	0.81	.42
- Education	-0.219	1.68	.09
- Cognitive Symptoms	-0.006	0.02	.99
- Fatigue Symptoms	-0.388	1.31	.19
- Physical Activity	0.035	0.14	.99
- Need for Cognition	-0.184	0.75	.46
- Experience of Pleasure	0.431	1.58	.11
- Depression Symptoms	0.208	0.52	.61
Linear reward sensitivity →			
- Age	-0.016	0.38	.71
- Education	0.091	1.84	.07
- Cognitive Symptoms	0.162	1.14	.25
- Fatigue Symptoms	-0.077	0.69	.49
- Physical Activity	0.198	1.89	.06
- Need for Cognition	0.289	3.01	.003**
- Experience of Pleasure	-0.192	1.83	.07
- Depression Symptoms	0.019	0.12	.90

Quadratic reward sensitivity →			
- Age	-0.004	0.33	.75
- Education	0.020	1.46	.15
- Cognitive Symptoms	0.038	0.96	.34
- Fatigue Symptoms	-0.003	0.09	.93
- Physical Activity	0.025	0.91	.33
- Need for Cognition	0.060	2.22	.03*
- Experience of Pleasure	-0.055	1.85	.06
- Depression Symptoms	-0.017	0.39	.70
- Age	-0.008	0.43	.67
- Education	0.026	1.15	.25
- Cognitive Symptoms	0.013	0.21	.83
- Fatigue Symptoms	0.039	0.77	.44
- Physical Activity	0.015	0.36	.72
- Need for Cognition	-0.080	1.87	.06
- Experience of Pleasure	0.044	0.95	.34
- Depression Symptoms	0.046	0.66	.51

Table S6. Results of the structural equation model: covariances between latent factors.

**Latent factors**	**Covariance**	***z*-score**	***p***
Cognitive symptoms ~			
Fatigue Symptoms	0.498	4.76	<.001***
Physical Activity	-0.052	0.71	.48
Need for Cognition	-0.246	3.29	.001**
Experience of Pleasure	-0.110	1.52	.13
Depression Symptoms	0.710	1.58	<.001***
Fatigue symptoms ~			
Physical Activity	0.125	1.61	.11
Need for Cognition	-0.137	1.84	.05
Experience of Pleasure	0.014	1.14	.83
Depression Symptoms	0.578	5.25	< .001***
Physical Activity ~			
- Need for Cognition	-0.120	1.52	.13
- Experience of Pleasure	-0.183	2.06	.04*
- Depression Symptoms	0.065	0.90	.37
Need for Cognition ~			
- Experience of Pleasure	0.300	3.59	<.001***
- Depression Symptoms	-0.263	3.63	<.001***

Experience of Pleasure			
- Depression Symptoms	-0.279	3.51	<.001***

Alternative versions of Figures 3, 4 and 5

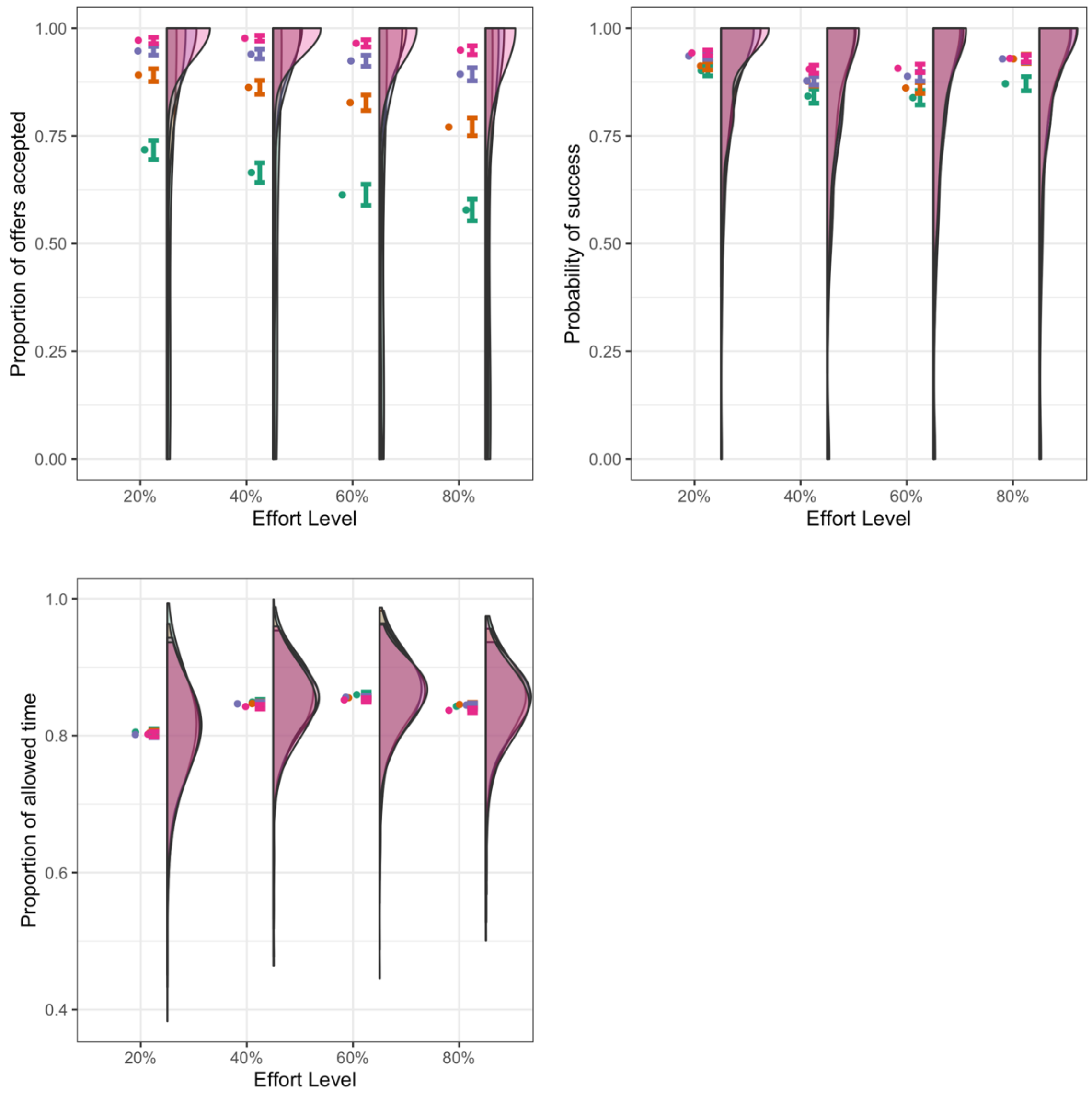

Figure S9. *Alternative versions of Figures 3, 4 and 5, without faceting.* (a) Proportion of offers accepted, (b) Probability of trials completed successfully, and (c) Completion time.

Sensitivity Analyses

In this section we repeat the modelling analyses using the two runner-up models, named Model 4 (vI + vR + vE) and Model 7 (vI + vR + vE + vE^2). First, the posterior parameter estimates are shown below in Figures S10 and S11. We can see that the intercept, reward and effort sensitivity parameter estimates are all substantially similar both across Models 4 and 9 and to those in the winning Model 9 (vI + vR + vR^2 + vE), shown in Figure 8 in the main text. Of note however is that while Models 4 and 7 had similar WAIC scores, Model 7 is less precise than Model 4 in its estimate of the linear effort sensitivity parameter; at the same time the quadratic effort sensitivity parameter both overlaps with zero and is highly colinear with the linear parameter (the correlation between the two is $r =$ -0.92, $p < .001$). Together this suggests that the linear and quadratic effort terms are redundant and trade off with one another. Nevertheless, focussing on the linear reward and effort sensitivity parameters we see, as with the winning Model 9, that these do not overlap zero, again according with the earlier ANOVA results).

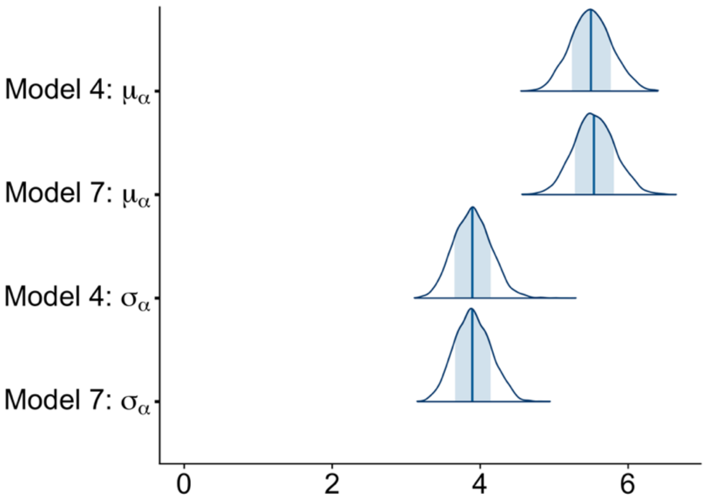

Figure S10. *Posterior distributions of the population-level intercept parameters in models 4 (vI + vR + vE) and 7 (vI + vR + vE + vE^2).* The vertical line indicates the mean of each distribution, and the shaded region the 66% quantile interval.

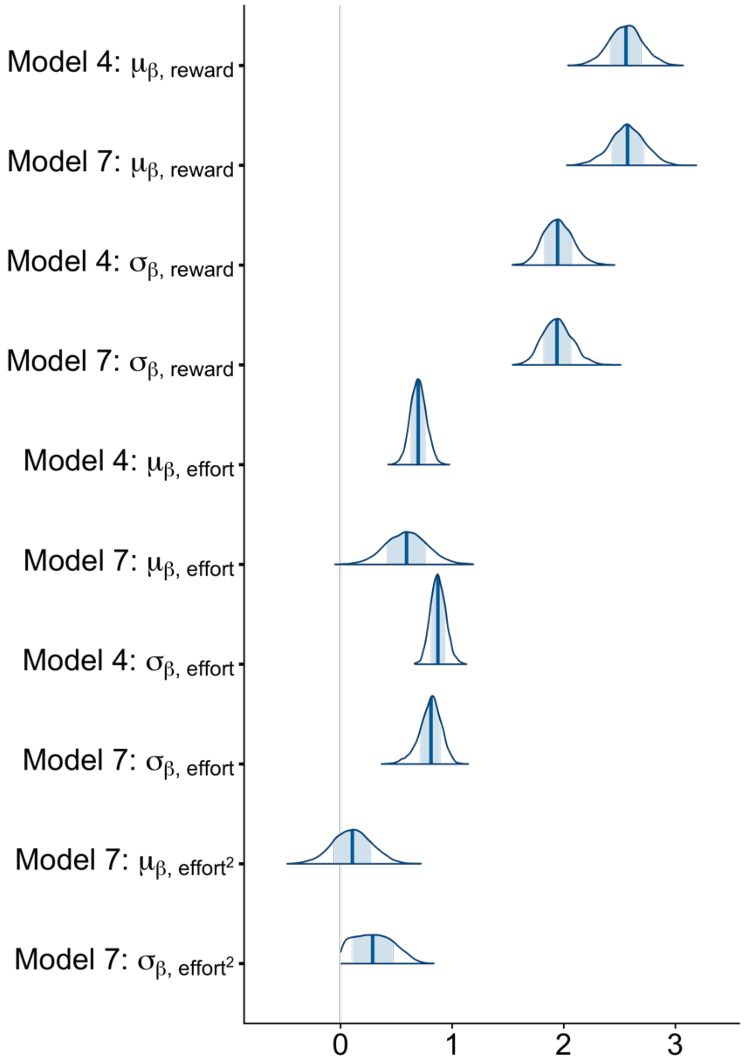

Figure S11. *Posterior distributions of the population level reward and effort sensitivity parameters in models 4 (vI + vR + vE) and 7 (vI + vR + vE + vE*^{2}*).* The vertical line indicates the mean of each distribution, and the shaded region the 66% quantile interval. The quadratic effort sensitivity parameter (Model 7: $\mu_{\beta,effort^2}$) substantially overlaps 0 and, additionally, there is a very large negative correlation between this and the model 7 linear effort parameter, $r =$ -0.92, $p < .001$, indicating substantial colinearity.

The posterior predictions from Model 4 (vI + vR + vE) and Model 7 (vI + vR + vE + vE^2) are shown in Figures S12 and S13. Again we see that both models produce very similar predictions, and both produce a reasonable overall fit to the data while still including substantial uncertainty about the behaviour of an individual participant. In this respect the two models are nearly identical to the winning model considered in the main text, Model 9.

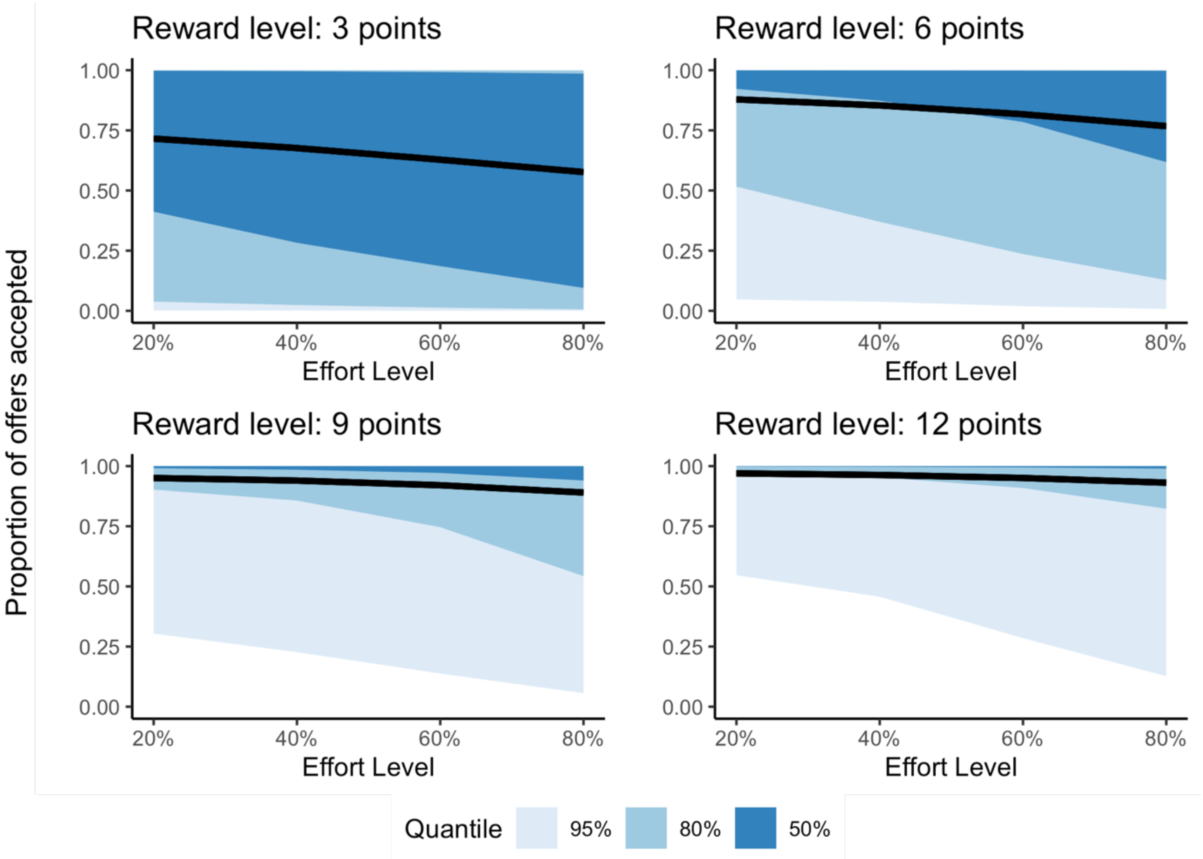

Figure S12. *Posterior predictions from Model 4 (vI + vR + vE)*. Plots show mean (black line) and posterior quantiles (95%, 80% and 50%) of the predicted probability of accepting an offer across each level of reward and effort. Note that these are predictions for simulated new participants and therefore incorporate uncertainty not just about the average effect of the manipulations in the population, but also about the behaviour of individual participants.

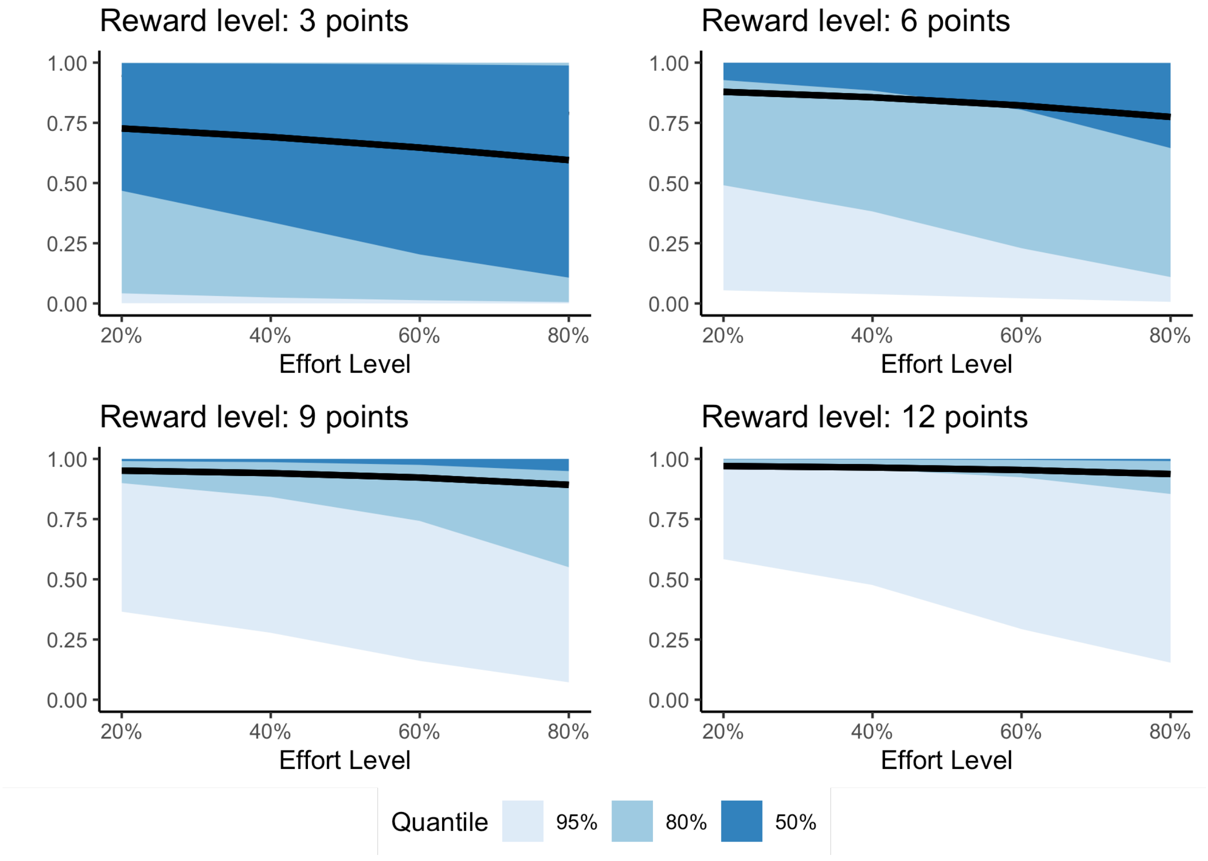

Figure S13. *Posterior predictions from Model 7 (*vI + vR + vE + vE^2). Plots show mean (black line) and posterior quantiles (95%, 80% and 50%) of the predicted probability of accepting an offer across each level of reward and effort. Note that these are predictions for simulated new participants and therefore incorporate uncertainty not just about the average effect of the manipulations in the population, but also about the behaviour of individual participants.

Finally, we examined the correlations between the participant-level effort sensitivity parameters (from both Model 4, vI + vR + vE, and Model 7, vI + vR + vE + vE^2) and the probability of success on the task (see Figure S12). Again mirroring the results with the winning Model 9 (vI + vR + vR^2 + vE), these correlations were not significantly different from zero (Model 4: $r(288) = 0.10$, $p = .09$; Model 7: $r(288) = 0.10$, $p = .07$), supporting our conclusion that effort sensitivity was not confounded by probability discounting.

Overall these sensitivity analyses support the conclusion that all of the three best performing models on WAIC score lead to very similar inferences, and therefore the results reported in the main text using Model 9 (vI + vR + vR^2 + vE) are robust.

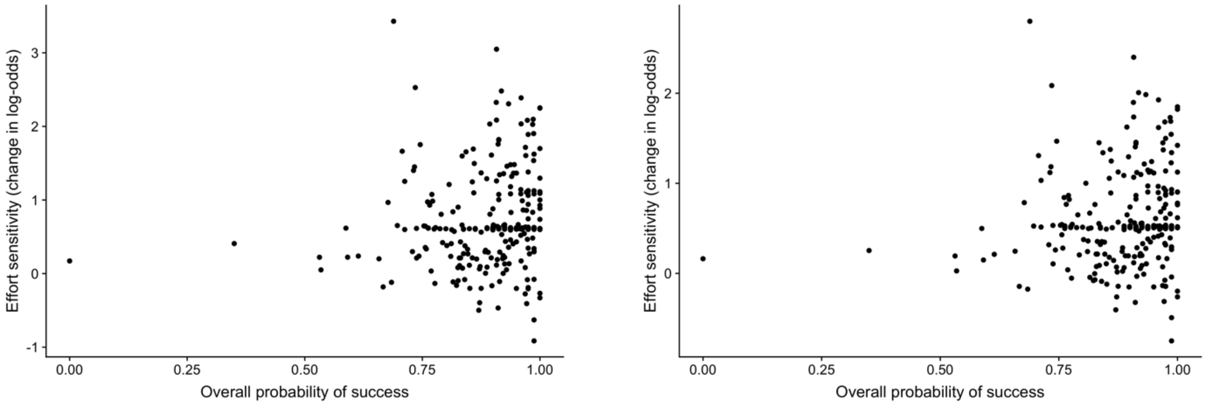

Figure S14. *Relationship between the probability of success and effort sensitivity*. Plots show (left) Model 4 (vI + vR + vE), and (right) Model 7 (vI + vR + vE + vE^2). The correlation was non-significant, implying that effort sensitivity is not confounded by probability discounting in this task.

Further tests of correlations between allowed time, success rate and completion time

Table S7. Cognitive Effort Task: Correlations between time allowed, success rate and completion time for each combination of reward and effort.

Correlation between time allowed and success rate		
**Reward: Effort**	**r**	**t-test**
3: 20%	$r = 0.07$	$t(253) = 1.15, p = .25$
3: 40%	$r = 0.14$	$t(245) = 2.20, p = .03$*
3: 60%	$r = 0.06$	$t(223) = 0.86, p = .39$
3: 80%	$r = 0.05$	$t(253) = 0.72, p = .47$

6: 20%	$r = 0.03$	$t(278) = 0.54, p = .59$
6: 40%	$r = 0.07$	$t(276) = 1.09, p = .28$
6: 60%	$r = 0.06$	$t(266) = 0.83, p = .41$
6: 80%	$r = 0.05$	$t(261) = 0.75, p = .45$
9: 20%	$r = 0.01$	$t(284) = 0.10, p = .92$
9: 40%	$r = 0.14$	$t(283) = 2.30, p = .02$*
9: 60%	$r = 0.07$	$t(276) = 1.22, p = .22$
9: 80%	$r = -.10$	$t(275) = 1.60, p = .11$
12: 20%	$r = 0.04$	$t(286) = 0.71, p = .48$
12: 40%	$r = 0.04$	$t(286) = 0.61, p = .54$
12: 60%	$r = 0.08$	$t(285) = 1.38, p = .17$
12: 80%	$r = 0.07$	$t(283) = 1.21, p = .23$
**Correlation between time allowed and completion time**		
**Reward: Effort**	**r**	**t-test**
3: 20%	$r = -.40$	$t(253) = 6.95, p < .001$***
3: 40%	$r = -.48$	$t(245) = 8.55, p < .001$***
3: 60%	$r = -.52$	$t(223) = 9.09, p < .001$***
3: 80%	$r = -.34$	$t(214) = 5.38, p < .001$***
6: 20%	$r = -.42$	$t(278) = 7.69, p < .001$***
6: 40%	$r = -.47$	$t(276) = 8.80, p < .001$***
6: 60%	$r = -.51$	$t(266) = 9.59, p < .001$***
6: 80%	$r = -.51$	$t(261) = 9.69, p < .001$***
9: 20%	$r = -.43$	$t(284) = 7.97, p < .001$***

9: 40%	*r* = –.59	*t*(283) = 12.2, *p* < .001***
9: 60%	*r* = –.55	*t*(276) = 10.9, *p* < .001***
9: 80%	*r* = –.52	*t*(275) = 10.0, *p* < .001***
12: 20%	*r* = –.45	*t*(286) = 8.49, *p* < .001***
12: 40%	*r* = –.52	*t*(286) = 10.4, *p* < .001***
12: 60%	*r* = –.57	*t*(285) = 11.8, *p* < .001***
12: 80%	*r* = –.51	*t*(283) = 10.0, *p* < .001***
